# As, Cr, Hg, Pb, and Cd Concentrations and Bioaccumulation in the Dugong *Dugong dugon* and Manatee *Trichechus manatus*: A Review of Body Burdens and Distribution

**DOI:** 10.3390/ijerph16030404

**Published:** 2019-01-31

**Authors:** Gabriel Núñez-Nogueira, Alejandra Pérez-López, Juanita María Santos-Córdova

**Affiliations:** Hydrobiology and Aquatic Pollution Laboratory, División Académica de Ciencias Biológicas, Universidad Juárez Autónoma de Tabasco, Carretera Villahermosa-Cárdenas Km. 0.5 S/N, Entronque a Bosques de Saloya, 86150 Villahermosa, Tabasco, Mexico; alee.libertad@gmail.com (A.P.-L.); santoscordovaj@gmail.com (J.M.S.-C.)

**Keywords:** Sirenia, toxic metals, dugong, manatee, accumulation, body distribution

## Abstract

The death of dozens of manatees *Trichechus manatus* recently in Tabasco, Mexico, has captured international attention. Speculation about possible causes include water and food contamination by metals. Although federal authorities have ruled out water chemical pollution, the cause of these deaths is still awaiting conclusive laboratory results. Present work seeks to summarize information currently available on non-essential metals and those of great toxicological relevance in Sirenia (dugongs and manatees), highlighting its body distribution, presence in blood, and its relationship with their geographical distribution, gender and age, whenever possible. This paper focuses on the five elements: As, Cr, Hg, Pb and Cd, which are commonly considered as threats for marine mammals and reported in Sirenia. Some of these metals (Cr and Cd) were thought to be related to the recent deaths in Tabasco. All five elements are accumulated by Sirenia at different levels. Metal presence is associated to their diet but does not necessarily imply adverse effects for dugongs and manatees. Toxicological aspects and the human consumption risk in case of any illegal or traditional consumption in some cultures are discussed. Important toxicological research areas that need to be addressed are highlighted.

## 1. Introduction

During the last five months, between May and September 2018, the death of dozens of manatees *Trichechus manatus manatus* in the municipality of Macuspana, Tabasco, Mexico, has captured attention at the national and international level [1,2,3,4,5,6]. It is a species with a vulnerable status on the Red List of Threatened Species [7], and is in danger of becoming extinct in Mexico [8]. Additionally, this species has local and regional importance [9]. Speculation about possible causes of these deaths include water and food contamination by metals, along with contamination by other toxic chemicals and microalgae [2,3,6,10]. Manatees can accumulate these substances, which can affect their health. The analyses of environmental samples revealed the presence of some metals in water and three in manatees. Chromium was found in one specimen, while cadmium was detected in a female and its foetus [11,12]. Although federal authorities have recently ruled out water pollution from hydrocarbons, pesticides and metals [13], the causes of these deaths remain unclear [3,4,14], and investigations are still ongoing. For this reason, it has become a priority to have ample scientific information available and published so that authorities, the scientific community, and the general public have sufficient knowledge to make the best decisions to ensure their protection. For this reason, the present work seeks to summarise the information currently available on non-essential metals and of those of great toxicological relevance in Sirenia (dugongs and manatees), highlighting the metals bodily distribution, presence in blood and its relationship with their geographical distribution, sex, and general toxicology while also considering their concentrations.

Aquatic contamination and presence of metals has been studied in different areas of southeastern Mexico [15,16,17,18], but few studies have focused on aquatic mammals throughout the whole country, particularly on manatees [15,19]. This becomes more relevant when we consider that Mexican and international laws protect these organisms, and there are massive deaths without a clear explanation in different scenarios, such as death by entanglements, collisions with vessels or algal blooms that have been observed in other geographical areas of importance for marine mammals, such as the Florida Peninsula [20,21,22], Brazil [23], Atlantic Ocean [24], China [25,26], Africa [27], South America [28], Russia [29,30], or United Kingdom and Europe [31,32,33,34,35,36,37,38]. However, despite its ecological importance, little is known about the regional concentrations of metals that could be considered typical or atypical in manatees. The effects or possible characteristics associated with their body presence and quantity, which may be present in one of the more iconic aquatic mammal species, such as *T. manatus manatus*, in tropical and subtropical waters needs to be determined.

**Figure 1:**
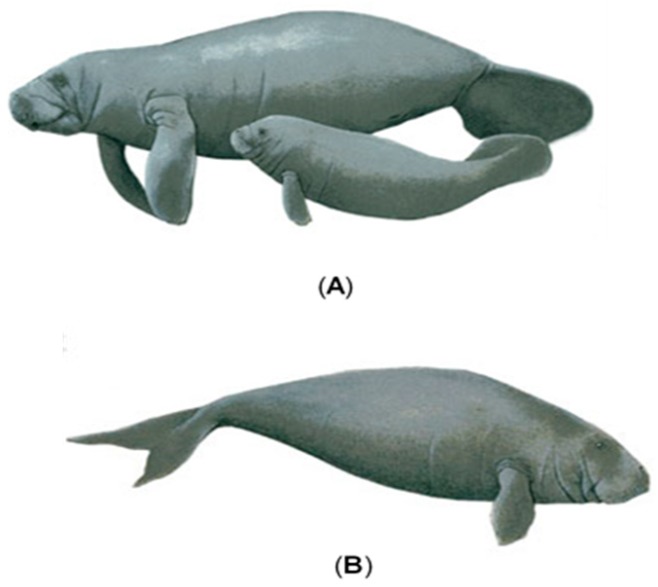
Representation of: (**A**) *Trichechus manatus* (manatee) and (**B**) *Dugong dugon* (dugong), taken from Save the Manatee Club [48].

Manatees are characterised by their differences from other aquatic or marine mammals. They are entirely aquatic and strict vegetarians that obtain their food from shallow waters from floating and submerged plants [39,40,41]. They are associated with rivers, lagoons and estuaries, as well as coastal and bay environments [42]. The existence of only two families of the order Sirenia, the family Dugongidae and Trichechidae is now recognised. The first consists of only one species *Dugong dugon* (Australia and Pacific Islands; Figure 1), while the second includes *Trichechus inunguis* (Manatee of Amazonia), *T. senegalensis* (West African Manatee), *T. manatus latirostris* (West Indian Manatee) and *T. manatus manatus* (Antillean Manatee; Figure 1) [41]; the latter is the one that inhabits Mexican waters. In the case of the *T. manatus manatus*, a recent mass mortality of organisms has been observed in Tabasco, and the official report states that 36 manatees have died between May and August 2018 [6], but it has been speculated that there are more than 60 dead organisms, which is alarming if we consider that in 2010 it was estimated that there were only about 1000–2000 specimens in the whole of Mexico [43,44]. In 2016, only 100 were considered to live in the State of Tabasco [45,46], although the National Commission for Biodiversity (CONABIO), recognises that there are no definitive population records in Mexico [47], and an updated official number is lacking.

Metals are among the most common contaminants in aquatic environments. Studies in aquatic mammals in general have focused on the importance of metals in their reproduction, decrease in their populations and increased mortality rates, as well as tolerance, which in some cases is higher than in other mammals [28,29,35,49,50,51,52,53,54,55,56]. The studies involve highly toxic metals, such as lead, cadmium or mercury, which generates a toxic response in aquatic mammals [50,57,58,59]. Due to different characteristics associated with longevity, low reproductive rate, sensitivity to anthropogenic activities and various pollutants, both inorganic (metals) and organic (hydrocarbons, pesticides, etc.), manatees can be considered as bio-monitors of the environment’s health status [60], along with other marine mammals [23,24,25,61].

## 2. Trace Elements Reported in Sirenia 

The Sirenia, due to its distribution and feeding characteristics, mainly inhabit shallow waters near the coasts, exposing them to polluted conditions and contact with human beings. Because of this interaction, efforts to understand the type of metals and their concentrations in either manatees and dugongs, have focused on 37 metals. Among them, it is possible to find essential metals. These actively perform or participate in physiological or metabolic cellular processes, in plants, animals or both. Also, non-essential metals, those that have no known biological function, can be found. Under these conditions, it is understandable that the body distribution of the trace elements will vary depending on the physiological conditions and needs of each organ or tissue. It is necessary to analyse the body distribution, which allows target organs or main body sites of accumulation to be identified as well as concentration amounts, that later could reach toxic levels that might compromise the health of the organs and the animal itself. 

Thirty-seven metals or minerals have been reported and are available in Sirenia scientific literature. These are: Ag, Al, As, B, Ba, Be, Ca, Cd, Cl, Co, Cr, Cu, Fe, Hg, K, Li, Mg, Mn, Mo, Na, Ni, P, Pb, Pt, S, Sb, Se, Si, Sn, Sr, Ti, Tl, U, V, W, Zn and Zr. Whole blood is the matrix with the highest number of trace elements analysed [39,62,63], followed by some studies in bones, liver, kidney and muscles [40,64,65,66,67,68,69]. This paper focuses on the five elements of significant toxicological relevance: As, Cd, Cr, Hg and Pb, which are considered a threat to marine mammals [70], have been reported in Sirenia, and have also been related to the recent deaths in Tabasco.

### 2.1. As (Arsenic)

Arsenic has been found mainly in the liver and muscle tissues of dugongs and manatees, and in the blood of *T. manatus*. Other tissues, such as kidney, bone, intestine and skin, have also shown the presence of arsenic, although reports of dugongs only found arsenic in the kidney and intestine, while in *T. manatus* it has been analysed more widely. 

Takeuchi determined the presence of arsenic in 28 different tissues from *T. manatus*, specifically urine, milk and in the contents of the digestive system [40] (Table S1). This manatee study is the most extensive, as far as the diversity of analysed tissues is concerned, both in wild animals and in carcasses. The highest mean concentration was observed in faeces and urine, Takeuchi [40] considered it was due to its characteristic of being a non-essential metalloid, this route of elimination has also been observed in other mammals, like rodents. Internally, the thyroid gland showed the highest concentration of arsenic. The author emphasised that although its effects on this tissue are not well known, its presence could be due to an antioxidant preventive response [40]. 

The blood matrix, plasma and erythrocytes have shown a range of mean concentrations of 10 to 30 μg/kg and from 100 to 1300 μg/kg, respectively (Table S2), considering food ingested as the main arsenic source in Florida [40]. For whole blood, the mean concentration ranged from 63 to 493 μg/kg [39,62]. The serum is the least studied fraction, with one of the lowest mean concentrations of arsenic (17.0 ± 20.0 μg/kg; [62]), which is consistent with the results observed by Takeuchi [40], where erythrocytes have a higher accumulation of arsenic. This accumulation capacity is relevant from the perspective of environmental monitoring if we consider that serum, unlike plasma or whole blood, is the fraction that remains after the separation of the coagulant components or once the blood has coagulated [71]. This occurs during the process of decomposing, as in the case of stranding, and therefore, serum is the most likely fraction to be obtained by monitoring marine mammals and recently deceased manatees. Plasma or blood would be of little use as a biomarker of exposure to arsenic or any other element; therefore, to ensure successful results, it would be advisable to take blood samples as soon as possible in those manatees that are already dead if arsenic is suspected to be involved. 

Regarding the liver, the concentrations ranged from 0.04 to 7.7 mg/kg (Table S3). The highest value has been observed in dugongs from the coasts of Queensland, Australia [65].

In the case of manatees, only one record has been observed in the liver in *T. manatus latirostris* from the Crystal River, Florida, with a mean concentration of 0.10 ± 0.01 mg/kg [40], 30 times less than observed in Australian dugongs (Table S3). Haynes et al. [65] did not find significant differences in stranded dugongs between 1993 and 2000 in different Australian sites but established that there was no apparent relationship between its concentration and toxic effects in stranded animals. O’Shea et al. [72], highlighted the need for chemical speciation analyses due to the presence of other methylated arsenic forms, which was observed in *D. dugon* from Japan in the early 80s [73]. O’Hara et al. [74] highlighted that different studies have shown that arsenobateine is the organic, non-toxic form of arsenic that is found in various marine mammal tissues, including blubber and skin in whales. Different forms of arsenic pass from soil or sediments to plants [75], but arsenic speciation has not been reported yet in Sirenia. Blubber and skin total arsenic concentration from *T. manatus* was <0.4 mg/kg (Tables S1 and S4), muscle (Table S5), kidney (Table S6) and brain (Table S7), correspond with low values observed in other marine mammals [74].

There have been some exceptions, particularly from Torres Strait, Australia, in the mid-90s, where four animals were analysed, and one showed 6.2 mg/kg of arsenic, while three other specimens had <0.08 mg/kg [67]. According to the Great Barrier Reef Marine Park Authority, more than 20 years ago, some areas were not considered as a potential hazard for local communities despite the presence of arsenic in local marine food. Liver and kidneys from dugongs as well as from other marine mammals are used in local meals and are generally not considered a risk for human consumption [74]. 

Only one Mexican study was found regarding arsenic in manatees sampled from Tabasco and Quintana Roo. Arsenic presence was detected in two different bones reported by Romero-Calderon et al. [19] in *T. manatus manatus* (Table S8). The concentration range was not detected up to 29.96 mg/kg in manatees from the Mexican Caribbean and the Gulf of Mexico. There was no correlation between concentrations depending on their origin (Tabasco or Chetumal), and more studies are recommended to contribute to assessing the toxicity of metals and tolerance of manatee in this regard in the region [19]. 

On the other hand, from a biological perspective of the Sirenia, no correlations have been observed between sex and concentrations of arsenic, but it seems a correlation exists with maturity status, at least in *D. dugon*, where mature individuals seem to accumulate more arsenic [65]; however, this has not been reported in more recent studies.

### 2.2. Cr (Chromium)

In Sirenia, chromium has been analysed primarily in bone and liver tissues, and there are some kidney, muscle and skin reports in *D. dugon* and *T. m. manatus* [15,19,64,65,66,76,77]. There are also records for Cr concentrations in blood of *T. manatus* and *T. m. latirostris* of Florida, USA, and Brazil [39,62,78].

Siegal-Willot et al. [62] reported the highest mean concentration of chromium in blood from *T. manatus* and *T. m. latirostris* combined (820.0 ± 80.0 μg/kg ww) from Belize and Florida, although three years later, Takeuchi et al. [39] observed concentrations lower than 100.0 μg/kg ww in samples from manatees from Belize (Table S2). In Florida, the mean chromium concentrations of 11.0 and 36.0 μg/kg ww were present and were higher than the values observed in Brazil (7.0, 9.0 and 10.0 μg/kg ww) in *T. manatus* [39,78] (Table S2).

As with arsenic, studies of Cr concentration in blood fractions revealed that erythrocytes and serum showed an increased accumulation in plasma, reaching values far above 100.00 μg/kg (Table S2). Chromium has been associated with a protein transporter in mammalian blood (chromoduline) and its distribution through the bloodstream and into the cellular interior by endocytosis through transferrin, as well as recognising it has an active role in the regulation of glucose and insulin [79,80]. 

In bone tissues, Nganvongpanit et al. [64] analysed different parts of the fangs from *D. dugon* (crown, root, superficial, intermediate and medial tusk), showing similar percentages among the tusk parts, ranging from 0.008 ± 0.001% in medial and intermediate tusk, up to 0.015 ± 0.026% in the crown tusk (Table S8).

Rojas-Mingüer et al. [77] revealed that chromium in the *T. m. manatus* cranial bone had a mean concentration of 2.9 ± 0.4 mg/kg ww in the Mexican Caribbean, which is less than the concentration observed in cortical bone, which obtained between 6.8–11.2 mg/kg ww, at least in the Gulf of México [19] (Table S8). 

With regard to target organs, the liver showed to be an important accumulation site, so there is data of Cr concentration in *D. dugon*, with a mean of 2.7 ± 4.0 and a range of 0.2–18.0 mg/kg ww; this is more than 30 times compared to the value reported 25 years before <0.2–<0.05 mg/kg ww in organisms from Queensland, Australia [65,66]. The highest concentration of chromium was found in the *D. dugon’s* liver (up to 18 mg/kg ww), followed by the cortical bone concentration from *T. m. manatus* in Mexican waters with reports of up to 11.2 mg/kg wet weight [19,65]. This metal’s presence in dugong’s liver is also higher than levels reported for muscle, bone, brain and kidney in the same species, with maximum concentrations of 0.5, <0.3, and <0.3 mg/kg dry weight, respectively [64,65,66]. On the other hand, Haynes et al. [65] observed that the mature *D. dugon* females had almost twice as much chromium in the liver 0.2–18.0 mg/kg ww. Regarding immature females, the authors observed a range of <0.02 to 10.2 mg/kg ww (Table S3).

Compared to other mammals, adults of the harbour porpoise *Phocoena phocoena* showed a significantly higher concentration of Cr, compared to juveniles [59], on the other hand, in *Kogia sima* a higher concentration in males has been registered [81]. In the dolphin *Stenella coeruleoalba,* a relationship was also found between body length and Cr concentration [82]. Although there is insufficient data in Sirenia, it is possible that the accumulation according to growth is increased by a process of bioaccumulation through age, i.e., longer exposure during the processes of sexual maturation. This suggests that bioaccumulation of chromium, especially in the dugong’s liver, could be related to the reproduction of females, increasing as maturation occurs. Studies in other mammals have shown chromium’s relationship with fertility (mainly in males) and pregnancies [80], as well as its requirement for the proper production of pre-lactation milk [83], could be similar in mature dugong females with breeding capacity and ageing, although more research is needed. Adults in *P. phocoena* showed a significantly higher concentration of Cr compared to juvenile individuals [59], which suggests a possible relationship in marine mammals in general. 

Data on the presence of chromium in kidney and muscle is limited to a couple of records in the *D. dugon* of Queensland, Australia, between 1974–1978, the first ranging from <0.02 to <0.03 mg/kg and the second from <0.03 to <0.50 mg/kg dw, for kidney and muscle, respectively [66]. In the case of brain tissue, it was observed by Denton et al. [66] that three males had metal concentrations below 0.3 mg/kg dw. Likewise, in the case of the skin, only one report of *T. m. manatus* of the Terminos Lagoon, Campeche, Mexico, with a mean of 0.783 and a range of 0.725–0.841 mg/kg ww was found [15]. In general, chromium is related to renal tissue as a result of blood filtration and body regulation through urine, reaching more than 60% chromium in mammals [80]. Its relationship with muscle tissue is not very clear but is recognised as one of the tissues with little or no accumulation of chromium [80]. Regarding the presence of chromium in skin, some authors have shown and used these levels of metals as an indirect indicator of internal levels in marine mammals. Sun et al. [26] explores this relationship, negative in the case of chromium, but not for other metals such as arsenic and mercury in the liver [26]. It is known that the skin has some resistance as a natural barrier to the passage of ions in mammals [84]; however, chromium has shown to be a highly toxic element for skin cells in whales. Hexavalent chromium is the most toxic form of metal due to its high cellular permeability, coupled with the most common form in which chromium is found in seawater, which has increased its importance and concern as a toxic agent for aquatic biota. Wise et al. [85] observed chromium concentrations in North Atlantic whale skin above those reported in South American whales, apparently associated with greater chromium contamination in northern waters, identifying cytotoxic and genotoxic effects for dermal cells, at least as observed in cell cultures. However, in Sirenia, more studies are required to determine their possible potential as a target tissue and its usefulness as an indicator of recent chromium exposure.

A comparison between chromium body distribution from dugongs and manatees allows a general pattern to be noted: higher concentrations in liver than in the kidney, which has also been shown in other marine mammals such as *Kogia sima* [81] or *Sousa chinsis* [26]. The only case where this distribution pattern was not followed and detected is that of the bottlenose dolphin (*Tursiops aduncus*), which recorded higher Cr concentration in the lungs (3.2 mg/kg dw), which was even higher than in the liver (2.2 mg/kg dw) and kidney (1.2 mg/kg dw) [27].

In Mexico, Romero-Calderón et al. [19] emphasised that the concentrations of Cr were higher in the coast of the State of Tabasco in *T. m. manatus* and highlighted the intensive use of fertilisers and pesticides, and oil extraction-related activities in the Gulf of Mexico [86], as the sources possibly associated with its environmental presence. 

It is important to highlight that no data was available for chromium in two of the manatee species: *T. senegalensis* and *T. inunguis* and the subspecies of *T. m. latirostris*.

### 2.3. Hg (Mercury) 

The first study on mercury in Sirenia was carried out on *D. dugon* with only two muscle tissue sampled in Indonesia [69], with values of 0.004 mg MeHg/kg ww (Table S5). Regarding *T. manatus* the first recorded report is from O´Shea et al. [68] with values below 0.02 and up to 0.02 mg/kg in manatee liver from Florida (Table S3). 

Mercury has not been detected or is rarely reported in some Sirenia tissues such as the intestine, gonad (Table S9), bone (Table S8), brain (Table S7) and skin (Table S4). Contrastingly, other tissues in which mercury has been reported more widely, are muscle and liver from dugons and manatees (Tables S3 and S5) as well as blood fractions in *T. manatus* and its subspecies *T. manatus latirostris* from Belize and Florida (Table S2).

The mean concentrations reported in the liver of dugons from Australia (the highest value observed in this organ) was 0.30 ± 0.29 mg/kg in mature organisms [65], while in immature organisms 0.09 ± 0.06 mg/kg (Table S3) was reported. On the other hand, in *T. manatus latirostris*, O´Shea et al. [21] reported a value of 0.12 mg/kg ww (0.54 mg/kg dw), which showed an increase of more than 9 times the observed amount 7 years later (1.11 mg/kg ww Haynes et al. [65]) in the same area of Florida (Table S3). All this information suggests an increase in the bioavailability of Hg in this region. 

The analysis of Hg in muscles are mainly reported in dugons; however, the concentrations are low, even sometimes less than the detection limits of the analytical technique employed. In the case of the manatee *T. manatus*, O´Shea et al. [68] reported a concentration of less than 0.02 mg/kg, so it has been considered that it poses no danger to those human populations consuming their meat because in humans, the recommended value of mercury in blood must be less than 10 μg/L [87]. In blood samples, the fraction with the highest observed mean value (20.0 ± 4.0 μg/kg) corresponded to erythrocytes of *T. manatus*, while the lowest value was also found in the same species, but in whole blood (Table S2), which is interesting since both samples came from the same site (Citrus County, Florida). This suggests that red blood cells are the main transporter of mercury in the bloodstream, at least in manatees, although it is possible that its persistence in the bloodstream is longer than 3 days, unlike other mammals, such as humans, or even lasts much longer in the blood because of its slower elimination rate [88].

Mercury values in Sirenia are much lower than those reported in cetaceans, dolphins and pinnipeds (Table S10), regardless of whether they come from coastal populations or not. 

The most studied tissue is the liver, followed by muscles and blood, the latter with only three reports: one from Antarctica and two from the United States. Values ranged from 0.099–0.658 mg/kg ww. The muscle tissue had a mean concentration of 0.06–4.44 mg/kg ww, while the liver showed the highest values: <0.01–2110.68 mg/kg ww (Table S10).

The highest value of Hg in the liver was reported for *Tursiops aduncus* from South Australia, with a mean concentration of 475.78 mg/kg ww [89]. Compared with the highest value found in *D. dugon* from Australia (0.30 mg/kg ww), this difference might be associated with the processes of biomagnification of the metal via ingestion of accumulating mercury in the dolphin’s diet, such as fish [56]. This is different from the vegetarian diet of the dugong, which probably has lower mercury content. In muscles, the highest value (4.44 mg/kg ww) belongs to *T. truncatus* of Portugal, while for *D. dugon*, the highest value was 0.005 mg/kg ww, while in the liver its range goes from <0.02 to 0.28 mg/kg [65,67,90,91,92,93]. No analysis reports were found for MeHg. 

Finally, it is in the blood tissue where the results are contrasting, with a higher value in Sirenia than in marine mammals (e.g., dolphins) as the *T. manatus* showed 9.0 mg/kg in blood, while for *T. truncatus* it was only 0.147 mg/kg [94]. This difference might be associated with the amount of exposure through the intake of Hg and type of food since Sirenia are herbivores and other aquatic mammals are carnivorous. That difference could account for a faster and higher accumulation of mercury through the processes of biomagnification associated with the type of diet. Food has an important role in Hg uptake, due to the amount of mercury in the muscle tissue of its meals. It is in an almost 100% methylated form and is bioavailable for its predators [95,96].

Another factor associated with the accumulation of methylmercury (the most bioavailable form of Hg) is its transfer to embryo seals through the placenta, and to a lesser extent, through the mother’s milk [97]. Reijnders [98] and Wagemann et al. [99], together with other authors, have documented that Hg crosses the placenta [100] and accumulates in the developing foetus. Therefore, the foetus is considered a “trap” or “sump” of mercury chelating the toxic element from the mother’s bloodstream [101]. The relationship between mercury and the development process of breeding is unknown, and differences are shown regarding the accumulation processes in the mother. In other mammals, mercury transfer has been well documented during pregnancy. In rats, the target organs are not always the same in the foetus as in the mother, changes in size or weight in the product are not always present and are related to exposure, despite maternal exposure to mercury [88]. In the case of humans, the transfer from mother to embryo has resulted in induction to abortion and to birth with various types of anomalies, such as cerebral, muscular or cognitive damage [88].

There are different variables related to the consumption and retention of mercury in marine animals. These include: diet, age, gender, health, proximity to urban areas and selenium residues, amongst others [50]. In the case of selenium, it is known that there is a relationship between Hg and other metals that contribute to the neutralisation of its toxicity, avoiding any possible adverse effects [102,103,104,105]. Therefore, it becomes important to consider analysing selenium in environmental monitoring studies when mercury presence is expected in Sirenia.

It has also been observed that the total mercury concentration in all mammalian tissues can show an increment depending on the age of the animal [99,106,107,108,109,110], especially in the liver [109,110,111,112]. This has been observed in *Phoca greonlandica* [107], *Delphinapterus leucas* [113], and *Monachus monachus* [114].

Regarding human interaction, we know that anthropogenic pollution generates differences in the presence of environmental mercury, compared with those areas that are not affected by human presence. Monteiro et al. [32] reported high mercury levels in *T. truncatus* (131.48 mg/kg ww), stressing that the values were similar or surpassed other animals in the Mediterranean, and indicating that these levels are due to anthropogenic sources of pollution. This result contrasted with the observations for the same species in Rio de Janeiro, Brazil, where Lemos et al. [23] reported a concentration of 42.63 mg/kg ww (Table S10), which turned out to be three times less than what the Mediterranean populations showed a few years later. Something similar could be happening with Sirenia living farthest from human settlements, or in protected areas, as noted by Takeuchi et al. [39], as well as by Takeuchi [40] for manatees in different areas of Florida. The highest concentration was found in dolphins (as was previously mentioned) in *T. aduncus,* with a value of 2110.68 mg/kg ww (Table S10). Leyvery et al. [89] discussed that these high concentrations could be related to the diet of *T. aduncus* (linked to benthic and sedimentary organisms), which is different from the concentrations found in the diet of pelagic dolphins like *T. truncatus*, supporting the idea of geographical availability of mercury as a cause of species accumulation. 

### 2.4. Pb (Lead)

Lead has been studied in tissues of both groups of Sirenia (dugongs and manatees), mainly in liver, bone, muscle, muscle plus fat, kidney, and to a lesser extent in skin and intestine [20,50,72]. Lead has also been evaluated particularly in whole blood, erythrocytes and serum from *T. manatus* and *T. m. latirostris* [39,40,62,63,78]. Its presence in Sirenia has been associated with the high presence of lead in aquatic plants [68], whose origin could perhaps be related to the high concentration of atmospheric lead that is detected in rain, as reported in Florida (0.44–1970 mg/g) [115].

The highest mean concentration of lead in blood was observed in *T. manatus* of Brevard County, Florida, in USA with 190 ± 2 μg/kg, and 4.0 ± 4.0 μg/kg in erythrocytes, followed by *T. manatus* from Brazil with 100.0 ± 66.0 μg/kg [39,78]. Interestingly, lower blood lead values were registered in *T. m. latirostris* from Crystal River, Florida, with 13.3 ± 3.0 μg/kg, followed by *T. manatus* from Belize with values of <50.0 μg/kg in whole blood and 30.0 ± 10.0 μg/kg in erythrocytes [39,63]. These differences suggest that there is no direct relationship between total lead in blood and lead in the erythrocyte fraction necessarily, so it is advisable to perform more studies on both fractions to determine the potential of erythrocytes as biomarkers of exposure.

The presence of lead in blood has also shown differences between wild organisms and in captivity. Organisms in captivity from Belize and Florida showed lower concentrations in whole blood and erythrocytes compared to wild manatees [39,63]. This appears to be related to a higher nutritional quality of the food provided in captivity, free of (or with less) lead content, compared to the vegetarian diet that they naturally find available in the wild. Similarly, this pattern was observed with other elements such as selenium, where Takeuchi et al. [39] highlights the need to monitor the presence of essential trace elements in the diets provided to manatees in captivity, which would help maintain their nutritional quality and would avoid metallic contamination.

Regarding liver distribution of lead in Sirenia, concentrations reached higher amounts in the species *D. dugon* (<0.08–3.08 mg/kg ww) from Queensland, Australia, compared with *T. m. latirostris* (0.1–5.1 mg/kg ww) from Caloosahatchee River, Florida [21,65]. This increased presence of lead in dugong’s liver is also associated with the age and maturity of the organisms. Particularly, Haynes et al. [65] observed higher concentrations (<0.08–3.08 mg/kg ww) in mature individuals than <0.08–0.85 mg/kg in immature individuals. Something similar was observed in the muscle of both *D. dugon* (<0.3 mg/kg dw) [66], as in kidneys of *T. m. manatus* (3.3–7.1 mg/kg dw) [68], which tend to be greater in older individuals. The relationship of metals with maturity in marine mammals has been observed in other elements such as Cd and Hg in cetaceans and pinnipeds [116]. These three elements are considered non-essential, so their bioaccumulation depending on the amount of exposure and maturity might be related to lower excretion rates than essential elements as they have been in aquatic animals [117,118,119,120] and this could be the cause of their greater presence in older individuals exposed to lead during their longer lifespan.

The size or length of the organisms could also be related to the concentration of the metal and not only to the exposure time and age. Vighi et al. [121] analysed fin whale bones (*Balaenoptera physalus)* from Spain and they found higher Pb concentration in foetuses than in adult organisms, with higher concentration in developing foetuses compared to more developed foetuses, suggesting this could be because the concentrations of Pb in bones are diluted according to the foetus growth and its development. Lead concentration has also been positively related to body length in *Kogia sima* (liver), *Phocoena phocoena* (liver) and *Tursiops truncatus* (body length as age approximation) [32,81,122].

In bones, lead has been analysed in *D. dugon* and *T. m. manatus* [19,64,76,77]. The concentrations reported reached 128 mg/kg ww in bone of *T. m. manatus* from Quintana Roo, Mexico [76], the highest reported in Sirenia. There are other data reported in bones of *T. m. manatus* (Table S8), which show a mean concentration of 41 mg/kg in organisms from Chetumal Bay, 14 mg/kg from the Gulf of Mexico and 11.2 mg/kg from other areas of the Mexican Caribbean [19,76,77]. In the case of dental bone, Nganvongpanit et al. [64] analysed lead distribution in different fang parts of *D. dugon* (crown, root, superficial, intermediate and medial tusk), finding similar percentages in the crown, root and superficial parts of the tusk (0.001 ± 0.001%), while in the middle and intermediate part of the fang, concentrations were below the detection limit (Table S8). Perhaps the fang surface is the oldest part and with higher amount of exposure and accumulation of Pb, unlike the middle and intermediate part of the fang, which is new growth and therefore has less exposure time to accumulate lead. However, the accumulation of lead varies depending on the type of tooth and the developmental stage during the exposure to metal in mammals (pup, young, and adult) [123,124,125], which generates variation and makes metal/age correlation unclear [124,125].

In the study of Romero-Calderon et al. [19], females of *T. m. manatus* exhibited a mean of 11.5 mg/kg lead in cortical bone, slightly lower than males (12.5 mg/kg); similarly by age, there was a difference in mean concentration in young (9.1 mg/kg ww) and adults (13.5 mg/kg ww), which again corroborates this type of correlation. 

The highest concentration of lead in tissues was observed in the bones of *T. m. manatus* from Quintana Roo Mexico with 128 mg/kg ww [76] and 41 mg/kg ww of *T. m. manatus* from Chetumal Bay, Mexico [77]. Despite this high value, lead concentration in bones from the same species from other coastal areas in southwestern Mexico showed a decreasing trend up to 10 times lower from 1999 to 2015 [19,76,77]. This could be related to the use of unleaded gasoline since the 1990s, which has reduced its atmospheric presence in the area [46]. This has also been observed in Florida and the Caribbean [115,126,127]. O’Shea et al. [72] pointed out that lead and copper concentrations in livers and kidneys of manatees have decreased since the 1970s. However, individuals of *T. m. latirostris* of the Caloosahatche River, showed slightly higher lead concentrations (0.44–5.1 mg/kg dw) than those reported seven years earlier for Crystal River individuals (1.8–4.4 mg/kg dw) [21,68]. The highest concentrations were found in *T. manatus* from the coasts of Crystal River, Florida, with values from 3.3 to 7.1 mg/kg, which suggests an accumulation unrelated to the changes of lead in gasoline, unlike what seems to have happened in Mexico. It was also observed that concentrations have tended to increase relative to those recorded at the beginning of the 80s by Denton et al. [66] (<0.1–<0.3 mg/kg) in Florida, to those reported 25 years later in the same site (<0.08–3.08 mg/kg) by Haynes et al. [65]. 

In dugongs, the presence of lead does not seem to have been affected or modified in Torres Strait, Australia, between the years 1993 and 1996, with values close to 0.07 mg/kg ww [67,82]. 

Comparing the presence of lead in Sirenia with other aquatic mammals, it is observed that lead concentration is mainly associated with bones in whales (e.g., *Balaenoptera physalus* and *B. acutorostrata*), which might be related to age, considering the possibility of it being a calcium-competitor [121]. The distribution in different tissues of whales showed a pattern of greater to lesser concentration as follows: bone > kidney > muscle > liver [128]. This same distribution was observed in the Pacific walrus (*Odobenus rosmarus divergens*) from Russia, where the highest concentration of Pb was detected in the bones (36.97 μg/g dw) followed by lung, kidney, muscle, seminal gland, heart, intestine, spleen and liver [30]. These distributions agree with the pattern observed in Sirenia, particularly with the main target organs in dugongs and manatees: bone, kidney and liver. Haynes et al. [65] found no difference in liver lead concentrations regarding sex, nor did they observe correlations between the cause of death of dugongs and concentrations of metal in the liver. 

The lead in muscle has been mostly analyse in *D. dugon* than in *Trichechus spp.*, of which there are no reports (Table S5). The mean concentration of lead in the muscles of *D. dugon* was higher in Sulawesi Island, Indonesia (0.25 mg/kg), and the lowest mean (0.035 mg/kg) in Torres Strait, Australia [66,67,69,82]. Miyazaki et al. [69] reported a mean of 0.25 mg/kg ww lead in the muscles of a mature 18-year-old female, greater than the one reported for an immature 7-year-old female (0.02 mg/kg ww). There are also some lead concentrations reports combining muscle plus fat from *D. dugon* of Torres Strait, Australia, with ranges from 0.02 to 0.03 mg/kg of lead [67,83], not dissimilar from values reported independently (Table S5).

In the kidney, lead concentrations have been studied in *D. dugon* from Australia and *Trichechus manatus* from Florida [66,67,68,82]. The second tissue with the highest concentration of lead was the kidney with 5.2 ± 1.0 (3.3–7.1) mg/kg dw in *T. manatus* from Crystal River, Florida [68], and the lead concentration in the liver ranged from 0.08 to 3.08 mg/kg ww of *D. dugon* in Queensland, Australia [65]. On the other hand, the concentrations of lead in skin showed records for *T. m. latirostris* of Florida and *T. m. manatus* from Campeche, Mexico [15,94]. The highest concentration was found in *T. m. manatus* from Terminos Lagoon, Campeche, Mexico with 0.265 (<L.D. (limit of detection) to 0.529 mg/kg), while in *T. m. latirostris* of Florida, this concentration was 0.036 ± 0.058 (0.006 to 0.178) mg/kg ww [15,94]. In tissues like skin, muscle, muscle plus fat, and intestine, the highest concentration of lead were 0.50, 0.04, 0.03 and 0.03 in mg/kg ww, respectively [15,67]. Gladstone [67] reported lead presence in the intestines from a *D. dugon* specimen from Torres Strait, Australia, with 0.03 mg/kg ww, so the lead study on this tissue should be expanded to further understand the distribution and absorption of lead along the intestine.

### 2.5. Cd (Cadmium)

Cadmium is one of the most dangerous metals for aquatic biota. Its presence and adverse effects are mainly associated with kidneys and bones, but the lung and liver are also linked to this metal accumulation and regulation of body burdens. Cadmium has been found mainly in the liver, dental bone, muscle, kidney, and some samples of brains in *D. Dugon*. In manatees, on the contrary, its presence has been analysed in blood fractions, bones, kidney and skin. No record was found during this review about cadmium in liver, brain and muscle of *T. manatus*, nor in blood fractions or skin in dugongs. 

In manatees, the concentration range for cadmium in blood goes from 0.4 to 8.2 mg/kg, while in serum and erythrocytes, it ranges from 0.1 to <10.0 mg/kg mean values (Table S2). Plasma is the fraction with the highest presence of cadmium, usually around 10 mg/kg in Sirenia (Table S2). Takeuchi [40] reported the highest concentration of cadmium in plasma with an average of 100 ± 50 mg/kg, which might be associated mostly to metallothionein regulatory proteins, as observed in other mammals [128,129]. A relationship of cadmium with metallothionein in marine mammals of up to 98% [130] has even been observed. These proteins are involve in chelating and reducing the availability of metals intracellularly, avoiding adverse effects at the cytoplasmatic level. However, although there is presence of these proteins in blood, there is evidence of the effects of exposure to cadmium and lead at the blood level on the antioxidant response in mammals, particularly in humans [131]. Due to this, there is the need to study in more detail these type of biomarkers in Sirenia blood to better understand the possible relationship between the cadmium present in plasma and the antioxidant response in blood.

In general, if we compared Cd concentrations reported with other metals studied here, cadmium and mercury are those that have shown less presence in blood fractions in Sirenia (Table S2). This same pattern has also been observed in other mammals such as *Mirounga leonina* [132] and *T. truncatus* [133]. 

Regarding body distribution, particularly in the liver, cadmium has only been reported in dugongs (Table S3), ranging from <0.1 to 58.8 mg/kg dw (or <0.005–32.5 mg/kg ww) [65,66]. Its presence in the liver of *D. dugon* is evident as the most concentrated metal compared to As, Cr, Hg and Pb (Table S3). Its presence has resulted in a positive correlation between the age of the organisms, as well as in the kidney [66] and the degree of maturity [65]. This type of response has also been detected in dolphins where the presence of cadmium in the liver is positively correlated with age [126]. Likewise, Lemos et al. [23] discusses the positive relationship between cadmium and length and age in other marine mammals, which occurs in several species. 

Denton et al. [66] also observed a relationship between the presence of cadmium in the liver and the sex of wild dugongs, concluding that there is a greater presence of cadmium in males than in females, which was not observed in other tissues such as dental bone. Yet, Denton et al. [66] did not detect metal correlation regarding sex in the case of stranding animals in Queensland, Australia. Haynes et al. [65] did not observe a relationship between cadmium concentration and the state of health, contrary to Denton et al. [66]. From that study, an association between different metals present in dugongs, including cadmium, and their death was not found. Similarly, O´Shea [68] considered that cadmium did not provide a risk to Florida manatee populations, at least in specimens studied during the 1970s and early 1980s. All these cases demonstrated that cadmium presence per se does not necessarily mean lethal effects or severe damage to the animal.

On the other hand, the presence of cadmium in the liver could affect the presence of other metals, such as copper, even at concentrations lower than those observed in other metals [134,135]. This is important if it is considered that according to Denton et al. [66], the dugongs ingest between 1 and 6 mg of cadmium per day through their food. If this intake and ingestion is continued for long periods, it could be considered that its effects on the metabolism and accumulation of metals like copper, coupled with a low presence of copper in food, would generate an imbalance in the levels of copper in dugongs, in correlation with cadmium assimilation efficiencies. This could occur in northern Australian areas where Dight and Gladstone [92] observed seasonal increases in the bioavailability of different metals, mainly cadmium, in areas inhabited by dugongs. On the other hand, the presence of cadmium is also considered to possibly be diminished by the presence and interaction with certain proteins and other metallic elements, or as mentioned earlier in the case of mercury, selenium seems to play a protective role against the toxic effects of cadmium as well [23].

The skin is another tissue where only two studies have found the presence of cadmium in manatees [15,63] and none in dugongs. Stavros et al. [63] found cadmium in skin in *T. m. latirostris* from Florida in a range of 0.005 to 0.067 mg/kg ww in eight individuals, while Benítez et al. [15] observed lower concentrations from undetected concentrations up to 0.032 mg/kg ww in *T. m. manatus* (Table S4, Supplementary Materials). The skin has been considered an excellent tissue for non-invasive sampling along with blood in marine mammals [24,133]. Stavros et al. [63], noted that the concentrations of cadmium in skin were lower than those observed in the liver and kidney of stranded animals in Florida than the data observed 24 years earlier by O´Shea et al. [68]. At the same time, Benítez et al. [15] detected a higher concentration of cadmium in concordance with the trophic level in samples of blubber and skin, although the authors do not specify the values for blubber or skin or if they used both combined. 

Cadmium appears to not be related with skin samples from manatees as mercury does in dolphins and seals [136,137], and low cadmium presence in skin has been observed in other marine mammals, such as *Phoca vitulina*, suggesting this tissue as not being suitable for metal monitoring [137]. However, zinc and nickel showed good results in dolphin skin [138]. This relationship seems to depend on species and metal in pelagic mammals [26]. However, recent studies found suitable usefulness of skin for Hg, Cd, and Se monitoring in *Stenella coeruleoalba* dolphins from the Mediterranean Sea [61], and *Neophocaena phocaenoides* porpoises from China [25], which supports the idea of using skin for monitoring, depending on the species involved.

Cadmium in muscle has only been reported in dugongs. Concentrations lower than 0.2 mg/kg dw or ww appear to be the common values, but only 31 specimens have been reported so far (Table S5). No data was found for muscle in manatees. A similar number of samples have been studied in kidneys from dugongs (Table S6), with the highest cadmium concentration being found in *D. dugon* from Queensland, Australia reaching 309 mg/kg dw [66]. Minor concentrations have been reported in manatees from Florida, where the highest concentration reported was 190 mg/kg dw, which was less than the mean value of 26 mg/kg dw (Table S6) for 36 specimens [68]. 

Kidneys, like livers, are the most common tissues analysed for cadmium accumulation in marine mammals. Its presence is commonly considered in kidneys as a result of cadmium regulation and excretion through urine, and temporary storage in a detoxified form bound to metallothionein (or competing with metals like selenium). There are other cytein-rich molecules involved in metals’ antioxidant responses as has been discussed previously in several marine mammals [31,61,66,130,139]. Denton et al. [66] reported the highest cadmium concentration in kidney (309 mg/kg dw in dugongs), suggesting an efficient tolerance to this non-essential metal. Compared with other marine mammals, this value was higher, as discussed by Takeuchi [40]. Normally, high cadmium concentration in marine mammals is related to diet. Predatory mammals using squid or invertebrates, rather than fish, as main food sources showed higher cadmium concentrations [78,96,126,140,141]. The vegetarian dugongs and manatees can only obtain minerals and metals by ingestion, so metal analyses in food contents became imperative for any environmental correlation between metal body burdens and pollution. 

Surprisingly, even when ingested food might be the main cadmium source in Sirenia, opposite to liver and kidney, intestine and brain are among those tissues poorly analysed for cadmium. No reports for manatees from Florida were found in this review. Only three specimens with concentration <0.3 mg/kg of cadmium in brains (Table S7) and one specimen with 0.09 mg/kg in intestine (Table S9; reported since 1980 [67]) were detected in dugongs. This last value appears to be lower to those reported in the intestines of walruses, for example [30]. Das et al. [130] discussed that metal uptake deficiency from diet could cause and increase metal absorption in the intestine as a result of the availibility of metal binding sites in intestinal epithelium when essential metals are poorly present in the food ingested, as suggested in dugongs [66]. This allows higher metal uptakes and absorption when essential metals show lower intake efficiency, or when inefficient capacity to regulate body metal concentration when metals are in excess in the diet, which might occurre in manatees [68,130]. Seagrasses from Florida have shown up to 10.7 mg/kg cadmium and 33.68 mg/kg copper concentrations, recently [142]. Both metals might be competing for intestinal binding sites, as suggested by Das et al. [130], reducing cadmium uptake in manatees, also as a result of fast food passage rates in the small and large intestine [40], which explains the lack of cadmium presence in this tissue compared to responses observed for essential metals like Cu or Zn [40]. Cu has already showed a negative correlation with Cd in liver of dugongs [66], supporting this idea.

Regarding cadmium presence in brain tissue, Gajdosechova et al. [136,140] highlighted that some transporters involved essential substances uptake by the brain, and might also be involved in toxic metals like cadmium or mercury uptake through the blood–brain barrier. It is considered that those metals already absorbed from the intestinal track enter the bloodstream and reach the central nervous system [143]. Time of exposure is also considered as an important factor, explaining why age showed good correlation with cadmium in the brains of pilot whales and might be affecting antioxidant responses [140]. The small sample size for Cd in brains of dugongs does not allow conclusive results, but a positive correlation between cadmium and age have shown that Cd in the liver and kidney are also highly concentrated in older specimens [66].

Cadmium is considered as one of the metals that most affects the bones, causing bone deformations, as well as the weakening and fracture of bones in mammals including humans [139,144]. Cáceres-Saez et al. [28] highlighted that metal analysis in marine mammals has been widely published but limited with respect to trace minerals in calcified tissue [13,16], mostly due to the difficulties in accessing marine mammal samples. Despite this, cadmium in bones has been reported in tusks from dugongs in Australia [64] and skulls, cortical, vertebrates, ribs and flipper bones from manatees in Mexican waters (Table S8). Cadmium in tusks of *D. dugon* does not exceed 0.02 ± 0.01%, regardless of the tusk part, section or type of tooth analysed (Table S8), whereas in the manatee *T. m. manatus*, the cadmium concentration ranges from 3 to 5 mg/kg ww, which is very similar to the different bones analysed, both in animals that inhabit the Caribbean and in the southern areas of the Gulf of Mexico (Table S8). However, when compared to other metals present, ribs showed a correlation between Cd-Cr, Cd-Mn, and Cd-Pb in *T. m. manatus* with different concentration on the skull [77].

In Mexican manatees, no correlation between cadmium concentration and age or sex has been observed in bones [19], in disagreement with Takeuchi et al. [39], who observed minor Cd in older organisms. Yet, positive correlations have been observed for other metals and ages in cetaceans or other marine mammals [121,126,130]. Perhaps these differences among manatees and other mammals are related to genetic and physiological features of each population, and the type of bones involved in the analyses, as discussed previously for lead, or even physiological or chemical features in different bones. Rojas-Mingüer [77] highlight that bones in manatees are denser, and discussed that calcium deposition is related with the type and amount of different metal uptake in bones. 

In marine mammals, cadmium has also shown differences in bone between different species of dolphins [126], mainly due to the availability of metals in the habitats that each species occupies, the coastal ones being the most affected concerning those inhabiting pelagic zones. 

Finally, it is recognised that differences among metals and other parameters like geographical location, and species involved, are closely related to sample sizes, age, maturity, diet composition, water sources, season of evaluation and anthropogenic factors as established by Siegal-Willow et al. [62]. For cadmium, different patterns have been detected in Sirenia, such as higher concentrations of Cd for captive and rehabilitated Sirenia than those in the wild and are also related to age in wild animals [39]. Similar differences regarding habitats were observed in whole blood cadmium, which was eight times higher in Brazilian manatees than those analysed in Florida [62] and compared to other mammals [78]. Cadmium has shown a higher concentration in organisms from the Mexican Caribbean compared to other aquatic mammals [77]. However, these same organisms did not show differences between offspring and adults concerning the presence of cadmium in bone and blood [77].

In dugongs, Cd concentrations correlated positively with age in both the kidney and liver [66], but not in dental bone and gender [64,65] or mature dugong livers, which have been related to anthropogenic pollution, but have not been related to mass mortality [65]. 

Likewise, Denton et al. [66] observed differences among cadmium and other metals in liver and kidney from different geographical areas, which is attributed to age differences between the groups analysed between Mornington Island and Townsville, Australia. However, they did observe significant differences between sex and cadmium in both tissues, being mostly accumulated in dugong males. 

## 3. Toxicological Considerations

Information and features related to toxicological effects of metals in marine mammals are limited [124,145] despite the ample literature regarding toxic metal studies in all marine mammal groups, forcing the extrapolation of terrestrial and human responses and health effects for comparison instead [124].

As it is possible to observe in this review, the five metals discussed here show different concentrations, and different body distribution in some cases, depending on the genus, age, size or weight, as well as the species involved, the geographical area and changes within decades. However, although the metals are present in the different organs and tissues, their presence could represent a possible risk or affect the proper functioning of target organs in the Sirenia, even if the concentration is below lethal levels reported in other mammals. It should be noted that during this review it was not possible to find reports of acute toxicity values or lethal concentrations for aquatic mammals. 

The concentrations and averages of the highest concentrations for As, Cd, Cr, Hg and Pb could compromise the proper functioning at different levels of organization, from the entire tissue or organ to a subcellular level (e.g., nuclear or cytoplasmic). In the particular case of the five metals studied here, the following observations have been made.

For arsenic, the range of concentrations in the different tissues and blood samples studied (plasma, blood, erythrocytes, serum, liver, kidney, muscle, bone, brain and intestine) in Sirenia range from 0.02 (skin) to 7.7 mg/kg dw (liver), and from 10 mg/kg (plasma) up to 1300 mg/kg (erythrocytes). The inorganic forms of arsenic are considered the most toxic and their effects range from biochemical or molecular alterations, affecting signaling pathways for activation or inactivation of proteins [146], to the generation of tumors and death in mammals such as humans [75]. The presence of arsenic in erythrocytes has already been observed in other mammals and has been implicated as the main mechanism of body distribution, mainly accumulating in the liver and kidneys [147]. Accumulation in the liver of Sirenia could mean an accumulation mainly in the insoluble fractions where As has been found to be mostly linked to molecules that are rich in thiolic groups, which chelate and store arsenic [147]. They are then removed from the liver towards the blood to be filtered later in the kidney, and then passing into the urine for excretion. 

The concentrations observed are below those detected in other marine mammals, at least for the most studied tissues, such as the liver, with a range from 0.001 to 8.7 mg/kg ww [124,148], the kidney with 0.03 to 47 mg/kg [124,148] and muscles with 0.02 to 1.98 mg/kg ww [148]. Blood is recognized as one of the fractions with the highest accumulation of arsenic, with 90% to 95% of metalloids in blood cells [149], although the 1300 mg/kg reported in erythrocytes of the manatee [40] exceeds the observed concentration in other mammals, such as seals, that have shown concentrations lower than 160 mg/L in pups [150]. The toxicological effects associated with arsenic are reported at 0.375 mg/kg in the liver, where it has been observed that it can lead to a reduction in the production or proliferation of lymphocytes in pinnipeds [151] and with an exposure of 25 mg/kg/day, which can induce oxidative damage in the kidney and brain of mammals [152]. It is not possible to rule out any adverse effects in Sirenia, although they are clearly highly tolerant to arsenic in their blood and body. 

In the case of Cd, the concentrations in Sirenia range from 0.005 (liver and skin) to 309 mg/kg (kidney). The highest concentration of cadmium in the kidney exceeds the observed values in dolphins from different regions around the world, which have shown a concentration no greater than 190 mg/kg [148,153,154]. The same pattern is observed for cadmium in the liver with up to 99 mg/kg in cetaceans [148,154]. For blood, plasma showed the highest values with 100 mg/kg in Sirenia, while whole blood did not reach more than 8.2 mg/kg (Table S2). Cadmium in marine mammals is within these ranges, and higher concentrations up to 581 mg/kg did not cause kidney damage or histopathological changes, as have been observed in seals [155,156]. However, renal fibrosis and lung fibromuscular hyperplasia have been related to liver cadmium in whales [157]. Kidney cadmium concentrations in Sirenia ranged from <0.1 to 309 mg/kg (Table S5). Interstitial fibrosis, functional involvement of the kidney proximal tubule and pulmonary hyperplasia have been associated with concentrations of cadmium in the kidneys of whales between 10 and 50 mg/kg ww [157]. Fujise et al. [158] highlighted that renal dysfunction can develop in marine mammals at very high concentrations (>800 mg Cd/kg dw). Also, cadmium, as well as arsenic, induces effects on the proliferation of lymphocytes [150], adrenal impairments and gonadic malfunction in seals [159]. Although, potential adverse effects of cadmium cannot be ruled out, marine mammals and Sirenia clearly tolerated higher cadmium concentrations than other mammals [155]. Also, its presence in pregnant females does not necessarily mean a risk for the foetus because the metal is not transferred through the placenta [124].

Chromium on the other hand, has been reported and detected in Sirenia from <0.1 (liver) to 820 mg/kg (whole blood). Its greatest presence has been associated with the liver and kidney of marine mammals, where it has shown concentrations between 0.006 and 9689 mg/kg [150]. Its presence in mammals has recently been associated with a relationship dependent on environmental concentration, which in turn is related to cytotoxic and genotoxic responses, with pulmonary, testicular and skin fibrosis in whales [160]. Also, it has been associated with hypersensitivity and autoimmune reactions, both in humans and marine mammals at blood concentrations as low as 20 μg/L [150].

The chemical form of chromium that is most commonly found in seawater is the hexavalent form, which crosses cell membranes more easily [85]. High concentrations of chromium in the environment has explained high concentrations in the fin whale *Balaenoptera physalus*, which has shown concentrations far above other whales and what is observed here in Sirenia. The metal ranges from 1.71 to 19.6 mg/kg of chromium in whale skin surpassed the maximum observed in Sirenia by a great amount (0.841 mg/kg, Table S3). Therefore, it is not ruled out that the cytotoxicity and genotoxicity may be developing in those whales living in waters with high hexavalent chromium content. It has also been seen that chromium hexavalent induces oxidative stress and apoptosis (cell death) in cutaneous fibroblasts of dolphins [161].

In the case of the kidney, concentrations of up to 23.51 mg/kg were observed in seals [124], resulting in measurements 100 times above that observed in dugongs, which is <0.3 mg/kg (Table S5). For all the above, it may be considered that Sirenia does not appear to be at immediate risk of chromium exposure, but more studies are currently needed to better define their effects and relation to anthropogenic emissions in the environment. 

Mercury, on the other hand, is another metal widely studied because of its high toxicity. Its presence in Sirenia ranges from 0.002 (muscle) to 20 mg/kg (erythrocytes). These values were already mentioned earlier to be much lower than those compared to predatory carnivorous mammal species, such as dolphins. The latter have shown concentrations of mercury between 0.21 to 1738 mg/kg in liver (approx. 1000 times higher than in Sirenia, Table S3), while in the muscle and kidney, the concentrations in cetaceans range from 0.09 (kidney) to 84.1 mg/kg (muscle), respectively. In Sirenia, they range from 0.002 to 0.05 mg/kg at the most (Table S6). The toxic effects associated with its presence are reported between 42.7 and 82.5 mg/kg ww in *Pagophilus groenlandicus*, which has shown to cause hepatitis, uremia, kidney failure, and even death [162]. On the other hand, in seals and dolphins, there have been reports of presence of mercury and methylmercury, which have been associated with various responses, such as renal damage by subcellular damage, lysosomal disorders, necrosis and fatty liver, and intestinal obstruction, at concentrations between 61 and 443 mg/kg ww in dolphins [163], whereas in calves of *Phoca vitulina*, exposure to mercury at concentrations as low as 0.5 mg/L for five days may cause inhibition in lymphatic proliferation [150]. At the testicular and suprarenal level, in vitro exposure of cell cultures showed alteration in the hormonal synthesis, compromising reproduction in seals [159] mainly by methylmercury. According to the Artic Monitoring and Assessment Program (AMAP) [164], the subclinical toxicological threshold concentration for marine mammals for mercury is 16.5 mg/kg ww in liver, a value quite above the values found in Sirenia’s liver (Table S3). 

Adverse effects can vary from changes in hormonal and enzymatic responses, behavioral changes and loss of sensory function, as well as appetite and weight loss [124]. The concentrations observed in Sirenia can be considered as innocuous and not able to induce toxic effects in dugongs and manatees. 

On the other hand, lead is also an element with a wide presence in the different tissues and blood of Sirenia. The lowest concentration reported is 0.02 mg/kg in muscle, ranging up to 190 mg/kg in the dugong’s blood. The presence of lead in blood is significant and may reflect effects on organs such as the liver. In *Tursiops truncatus*, concentrations of 84 mg/kg in liver and 0.66 mg/kg in blood have been detected, which have been related to hepatocellular hemosiderosis, hyperplasia, hypertrophy, renal disease and calcium deficiency among others [165]. Likewise, exposure to 50 mg/L of lead in blood has also been shown to induce reductions in lymphatic proliferation [166]. 

The toxic threshold levels for Pb have been established at 9.6 mg/kg ww in hepatic tissue and 21.6 mg/kg ww for kidney for other mammals [167]. Considering these reference values, lead has shown to be well below these numbers, with a maximum value of 5.1 mg/kg in the manatee’s liver (Table S3), while in the kidney, the highest concentration found was 7.1 mg/kg, which suggests no issues caused by lead in Sirenia, although we cannot disregard the need for more studies on these metals and their effects under chronic exposure conditions, as well as the effects at the biochemical, physiological and metabolic levels.

## 4. Human Consumption Risk

A final important aspect to consider is that the Sirenia, despite being a group of protected species in different countries where they inhabit, are still subject to illegal hunting for meat consumption, with its consumption is still recognized even in the twentieth century [168,169,170]. The per capita consumption of this meat could pose a risk to humans, depending on the metal consumed, the concentration of the metal involved and the average annual intake rate of meat per person according to the geographical area. Calculating the estimated dietary intake (EDI) according to Atique Ullah et al. [171], and considering the higher metal concentration reported (or higher metal concentration mean) in Sirenia meat, it is possible to observe in Table 1, that only arsenic came close to the reference value of possible risk for ingestion of meat. This result is based on the estimated daily intake of dugongs’ meat, reaching an EDI of 0.25 mg/day/person considering that the highest average concentration reported in dugongs was 3.13 mg/kg [67] for an average 60 kg adult’s body weight [171] and considering an average meat intake of 0.08 kg/day [172]. According to the FAO/WHO Joint Committee of Experts for Food Additives, the daily allowance for arsenic is 0.20 mg/day/person [173]. These EDI values mean that the concentration reported in 1996 in Australia was 1.9 times higher than the current recommended daily intake value (Table 1). The other four metals showed EDIs values below the intake recommended by the joint FAO/WHO Committee for Cd, Hg and Pb [174,175,176] and Cr [177]. All five concentrations obtained and analysed from no more than 41 individuals of Sirenia (Table S5), and available in the scientific literature, do not represent an immediate risk for human consumption, and agree with the results observed by Gladstone [67], who reported in 1996 the lack of human risk for consumption of dugong meat in Australia. The data suggests that if illegal consumption of meat, either from dugong or manatee, develops in local communities in Australia, USA, or Mexico, it will not be a risk from a toxicological perspective. However, more information and studies on the presence of As, Cd, Cr, Hg and Pb in Sirenia meat, as well as the average human intake of other Sirenia organs per year per person, are required to assess the risk of consumption of these organs and metals more accurately and extensively.

## 5. Conclusions

Sirenia accumulates and acquires non-essential metals and highly toxic concentrations of As, Cr, Hg, Pb and Cd. Both manatees and dugongs show body distributions depending on the metal involved, and their relationship with different biological aspects like growth, distribution and sex. For arsenic, its presence is associated to liver, muscle, blood, kidney, intestine and thyroid in manatees. Chromium has been observed in bones, liver, kidney, muscle and skin. Mercury has been evaluated in muscle and liver. Its presence is associated especially with its diet and it is highly considered to be accumulated, together with cadmium, even more so than in other mammals, without presenting evident adverse effects. Lead has been present in liver, bone, muscle and kidney. Its presence has been greater in dugongs than in manatees, and bone and skin in Mexican manatees have shown to be the highest in that group of Sirenia. Fangs have shown to be useful for the study of lead. Cadmium is mostly accumulated in kidney and bone, followed by liver in Sirenia. Depending on the marine mammal species, the skin can be an interesting tissue for environmental monitoring of cadmium. 

Regarding the correlation of metals with biological aspects in Sirenia, there is a correlation observed between maturity and arsenic (dugongs), but chromium needs more studies that relate to its presence with age and maturity in Sirenia, especially any possible maternal–foetal transfers. Lead has also been associated with age and maturity in these mammal groups. The geographical area is mainly related to the presence of lead as has been observed in other marine mammals. Cadmium has been correlated between the sex and the age of Sirenia, at least in the kidney and liver, and has shown greater presence in captive organisms.

Sirenia appears to tolerate higher metal concentration without toxicological response as in terrestrial mammals, but some concentrations recorded in this review are comparable to those reported in other marine mammals, and therefore, an adverse effect of metals on the target organs of the Sirenia cannot be ruled out. Regarding Sirenia´s meat consumption by humans, the five metals analysed do not appear to present a risk. 

An important area that needs to be studied are arsenic concentrations. Its speciation and biomarkers are associated in the dugong’s blood and arsenic in the liver of the manatee. Chromium on the other hand needs to be evaluated in the dugong´s blood and in the skin of the manatee and further studies in the muscles and kidneys of Sirenia. Mercury needs to be assessed in blood and its relationship with selenium in general in manatees and dugongs. On the other hand, lead and biomarkers associated with its exposure need to be studied, comparing wild and captive animals. Lead in muscle and intestine also need to be analysed. In the case of cadmium, there must be studies of the liver, brain and muscle of the manatee, as well as the blood and skin in dugongs, and increased metal studies in the muscles and brain. Studies of antioxidant biomarkers and cadmium in blood are needed for both manatees and dugongs. 

It is important to develop studies of these five metals reviewed here, in *Trichechus senegalensis*, *T. inunguis* and *T. manatus latirostris,* due to the lack of information available. In general, Sirenia shows a different accumulation and body distribution for some metals, compared to other mammals, so its presence per se does not necessarily imply a toxic or adverse effect for the species involved, as cadmium, chromium or arsenic concentrations might suggest in some cases.

## Figures and Tables

**Table 1:** Maximum metal concentration (mean or single reported value) in the muscle of Sirenia and dietary intake of metals via meat consumption by an average person (60 kg body weight).

Metal	Species	Maximum Metal Concentration in Muscle of Sirenia (mg/kg)	Weight of Meat Consumed by an Average Person by Country (kg/Day/Person)	Estimate Daily Intake ^d^ (EDI; mg/day)	Recommended Daily Dietary Allowance (mg/Day/Person)
As	*Dugon dugong*	3.13 [67]	0.080 ^a^ (Australia)	0.250	0.200 [173]
	*Trichechus manatus*	0.10 [40]	0.080 ^b^ (USA) 0.042 ^c^ (Mexico)	0.008 0.004	
Cd	*Dugon dugong*	0.19 (max. <0.20) [66]	0.080 ^a^ (Australia)	0.015	0.001 [174]
Cr	*Dugon dugong*	0.49 (max. <0.50) [66]	0.080 ^a^ (Australia)	0.039	0.200 [177]
Hg	*Dugon dugong*	0.01 [90]	0.080 ^a^ (Australia)	0.0008	0.030 [175]
	*Trichechus manatus*	0.019 (max. <0.02) [68]	0.080 ^b^ (USA) 0.042 ^c^ (Mexico)	0.0015 0.0008	
Pb	*Dugon dugong*	0.49 (max. <0.50) [66]	0.080 ^a^ (Australia)	0.039	0.200 [176]

^a^ According to The Australian Red Meat and Livestock Industry [172]. ^b^ According to Daniel et al. [178].^c^ According to Consejo Mexicano de la Carne [179]. ^d^ Calculated according to Atique Ullah et al. [171].

## Supplementary Materials

The following are available online at http://www.mdpi.com/1660-4601/16/3/404/s1, Table S1: Arsenic concentration (mean ± standard error and range, mg/kg; wet weight) in different body tissues and contents from *Trichechus manatus manatus* carcasses, opportunistic sampling; *n* = sample size, Table S2: Toxic metal concentrations (mean ± standard deviation; μg/kg, wet weight; *n* = sample size) in the blood fraction of manatees *Trichechus manatus*, Table S3: Toxic metal concentrations (mean ± standard deviation and range; mg/kg) in liver of dugongs and manatees from Australia and Florida, Table S4: Toxic metal concentrations (mean ± standard deviation and range; mg/kg; wet weight) in the skin of manatees, Table S5: Toxic metal concentrations (mean ± standard deviation and range; mg/kg) in the muscle of dugongs and manatees around the world, Table S6: Toxic metal concentrations (mean ± standard deviation and range; mg/kg) in the kidney of dugongs and manatees around the world, Table S7: Toxic metal concentrations (mean ± standard deviation and range; mg/kg) in the brain of dugongs and manatees, Table S8: Toxic metal concentrations (mean ± standard deviation and range; mg/kg) or percentage in bone of dugongs and manatees, Table S9: Toxic metal concentrations (mean ± standard deviation and range, or unique value reported; mg/kg; wet weight) in intestine and gonads of dugong and manatees, Table S10: Concentration of mercury (mean ± standard deviation and range, when applicable; mg/kg) in marine mammals around the world.
