# Supplementary material for: As, Cr, Hg, Pb, and Cd Concentrations and Bioaccumulation in the Dugong Dugong dugon and Manatee Trichechus manatus: A Review of Body Burdens and Distribution
Source: Int J Environ Res Public Health. 2019 Jan 31;16(3):404. doi: 10.3390/ijerph16030404 (PMC6388294; doi:10.3390/ijerph16030404)
Supplement: Supplementary file 1 [file ijerph-16-00404-s001.zip › ijerph-389570-suppl xml-1.docx]

**Table S1.** Arsenic concentration (mean ± standard error and range, mg/kg; wet weight) in different body tissues and contents from *Trichechus manatus manatus* carcasses, opportunistic sampling; *n* = sample size (modified from Takeuich [1]).

| **Tissue** | **Concentration** | ***n*** | **Tissue** | **Concentration** | ***n*** | **Contents** | **Concentration** | ***n*** |
| --- | --- | --- | --- | --- | --- | --- | --- | --- |
| Thyroid | 0.3 ± 0.1  (0.01–0.7) | 5 | Stomach | 0.04 ± 0.004  (0.03–0.04) | 2 | Milk | 0.02 ± 0.002  (0.01–0.02) | 4 |
| Thymus | 0.1 ± 0.02  (0.01–0.1) | 7 | Duodenum | 0.1 ± 0.03  (0.01–0.2) | 9 | Stomach contents | 0.03 ± 0.02  (0.01–0.1) | 2 |
| Lung | 0.1 ± 0.01  (0.01–0.1) | 9 | Duodenum horns | 0.01 | 1 | Duodenum contents | 0.01 | 1 |
| Cardiac gland | 0.01 ± 0.01  (0.002–0.03) | 2 | Cecum | 0.1 ± 0.01  (0.03–0.1) | 6 | Cecal contents | 0.03 ± 0.02  (0.02–0.10) | 2 |
| Heart | 0.04 ± 0.01  (0.02–0.1) | 6 | Cecal horns | 0.10 ± 0.02  (0.002–0.10) | 7 | Faeces | 0.8 ± 0.1  (0.1–3.4) | 23 |
| Spleen | 0.05 ± 0.02  (0.03–0.1) | 4 | Colon | 0.04 ± 0.02  (0.01–0.10) | 3 | Urine | 0.2 ± 0.1  (0.0–0.8) | 19 |
| Gall bladder | 0.1± 0.1  (0.01–0.1) | 2 | Ovary | 0.10 ± 0.03  (0.02–0.10) | 2 |  |  |  |
| Pancreas | 0.03 ± 0.01  (0.01–0.1) | 7 | Uterus | 0.04 | 1 |  |  |  |
| Blubber | 0.1 ± 0.02  (0.02–0.2) | 7 | Seminal vesicles | 0.01 | 1 |  |  |  |
| Bladder | 0.10 ± 0.04  (0.01–0.10) | 2 | Testes | 0.02 | 1 |  |  |  |

**Table S2.** Toxic metal concentrations (mean ± standard deviation; μg/kg, wet weight; *n* = sample size) in the blood fraction of manatees *Trichechus manatus*.

| **Fraction** | **Species** | **As** | ***n*** | **Cd** | ***n*** | **Cr** | ***n*** | **Hg** | ***n*** | **Pb** | ***n*** | **Location** | **Reference** |
| --- | --- | --- | --- | --- | --- | --- | --- | --- | --- | --- | --- | --- | --- |
| Whole blood | *T. manatus* | 300.0 ± 20.0 | 77 | 2.0 ± 0.2 | 75 | 11.0 ± 1.0 | 29 | <0.6 ± 0.1 | 20 | 30.0 ± 10.0 | 20 | CCF | [2] |
|  |  | 170.0 ± 20.0 | 20 | 3.0 ± 0.5 | 20 | 36.0 ± 2.0 | 10 | 9.0 ± 1.0 | 20 | 190.0 ± 2.0 | 14 | BCF |  |
|  |  | 190.0 ± 80.0 | 7 | 0.4 ± 0.1 | 7 |  |  | 1.5 ± 0.5 | 7 | 31.0 ± 9.0 | 7 | CF |  |
|  |  | 200.0 ± 20.0 | 14 | 1.0 ± 0.1 | 14 |  |  | 1.0 ± 0.3 | 13 | 9.0 ± 1.0 | 14 | CHF |  |
|  |  | 430.0 ± 70.0 | 33 | 4.0 ± 0.3 | 33 | <100.0 | 33 |  | 33 | <50.0 | 33 | BE |  |
|  |  | 63.0 ± 11.0 | 24 | 3.4 ± 0.4 | 24 | <100.0 | 24 |  | 24 | <50.0 | 14 | BE * |  |
|  |  |  |  | 1.7 ± 1.1 | 8 | 10.0 ± 3.0 | 8 |  |  | 50.0 ± 20.0 | 8 | PB | [3] |
|  |  |  |  | 1.9 ± 1.0 | 4 | 7.0 ± 3.0 | 4 |  |  | 43.0 ± 7.0 | 4 | AB |  |
|  |  |  |  | 8.2 ± 14.5 | 4 | 9.0 ± 2.0 | 4 |  |  | 100.0 ± 66.0 | 4 | PAB |  |
|  | *T. manatus latirostris* | 342.0 ± 44.0 | 8 | 1.0 ± 1.0 | 8 |  |  | 8.0 ± 17.0 | 7 | 13.0 ± 3.0 | 8 | CRF | [4] |
|  | *T. manatus* + *T. manatus latirostris* | 493.0 ± 250.0 | 45 | 1.0 ± 0.0 | 45 | 820.0 ± 80.0 | 45 | 1.01 ± 1.00 | 45 | 52.0 ± 110.0 | 45 | B/F | [5] |
| Plasma | *T. manatus* | 30.0 ± 10.0 | 40 | 100 ± 50 | 39 | 10.0 ± 1.0 | 31 |  |  |  |  | CCF | [1] |
|  |  | 10.0 ± 2.0 | 10 | <10.0 | 10 | 0.1 ± 0.1 | 3 |  |  |  |  | BCF |  |
|  |  | 10.0 ± 2.0 | 16 | <10.0 | 16 | 20.0 ± 10.0 | 16 |  |  |  |  | BE |  |
|  |  | 10.0 ± 2.0 | 8 | <10.0 | 8 | 10.0 ± 3.0 | 8 |  |  |  |  | F * |  |
| Erythrocytes | *T. manatus* | 500.0 ± 40.0 | 67 | 2.0 ± 0.1 | 64 | 10.0 ± 2.0 | 51 | 20.0 ± 4.0 | 64 | 40.0 ± 10.0 | 51 | CCF |  |
|  |  | 300.0 ± 30.0 | 11 | 0.1 ± 0.1 | 11 | 4.0 ± 0.4.0 | 3 |  |  | 4.0 ± 4.0 | 3 | BCF |  |
|  |  | 400.0 ± 100.0 | 10 | 1.0 ± 0.1 | 7 | 10.0 ± 9.0 | 8 | 3.0 ± 1.0 | 8 | 100.0 ± 20.0 | 8 | CCF |  |
|  |  | 300.0 ± 30.0 | 9 | 1.0 | 9 | 1.0 ± 0.3 | 9 | 2.0 ± 0.2 | 9 | 30.0 ± 5.0 | 9 | CCF |  |
|  |  | 1300.0 ± 300.0 | 15 | <10.0 ± 0.02 | 15 | 100.0 ± 30.0 | 15 | <10.0 ± 0.004 | 15 | 30.0 ± 10.0 | 15 | BE |  |
|  |  | 100.0 ± 20.0 | 9 | <10.0 ± 10.0 | 9 | 70.0 ± 20.0 | 9 |  |  | 20.0 ± 4.0 | 9 | F * |  |
| Serum | *T. manatus* + *T. manatus latirostris* | 17.0 ± 20.0 | 45 | 1.0 ± 0.0 | 45 | 386.0 ± 30.0 | 45 | 1.0 ± 0.0 | 45 | 3.0 ± 5.0 | 45 | B/F | [5] |

* Cautivity. CCF = Citrus County, Florida: USA; BCF = Brevard County, Florida: USA; CF = Collier Florida: USA; CHF = Charllot Florida: USA; BE = Belize; B/F = Belize and Florida; PB = Pernambuco, Brazil; AB = Alagoas, Brazil: PAB = Parabai, Brazil; CRF = Crystal River, Florida: USA; F = Florida.

**Table S3.** Toxic metal concentrations (mean ± standard deviation and range; mg/kg) in liver of dugongs and manatees from Australia and Florida.

| **Species** | **Location** | **As** | **Cd** | **Cr** | **Hg** | **Pb** | ***n*** | **STW** | **Reference** |
| --- | --- | --- | --- | --- | --- | --- | --- | --- | --- |
| *Dugong dugon* (Australia) | Queensland coast | 3.0 ± 1.9  (0.45–7.7) | (<0.005–32.5) | 2.7 ± 4.0  (0.2–18) | 0.3 ± 0.29  (0.05–1.11) | (<0.08–3.08) | 18 ^a^ | W | [6] |
|  |  | 2.1 ± 1.6  (0.04–5.3) | (<0.005–3.0) | (<0.2–10.2) | 0.09 ± 0.06  (0.04–0.28) | (<0.08–0.85) | 20 ᵇ |  |  |
|  |  |  | (<0.1–58.8) | (<0.2–<0.5) |  | (<0.1–<0.3) | 42 | D | [6] |
|  | Northern Territory |  |  |  | 0.05 |  | 2 | W | [7] |
|  | McArthur River | (0.2–0.8) | (10.0–15.0) |  | (<0.02–0.03) | (0.17–0.42) | n. s. | D | [8] |
|  | Northern, Territory |  | 16.4 |  |  |  | 1 ^c^ | D | [9] |
|  |  |  | 36.6 |  |  |  | 1 ᵈ | D |  |
|  | Torres Strait | 0.23 | 4.90 |  | 0.04 | 0.05 | 1 | W | [10] |
|  | Torres Strait: Great Barrier reef | 0.27 ± 0.11 (0.18–0.40) | 6.43 ± 2.74  (4.8–9.6) |  | 0.03 ± 0.01  (0.02–0.04) | 0.08 ± 0.029  (0.05–0.10) | 3 | W | [11] |
|  |  | (0.26–2.00) | (0.44–54.00) | (<0.1–2.9) | (<0.005–0.22) | <0.04 | 36 | D | [12] |
| *Trichechus manatus latirostris*  (Florida) | Caloosahatchee River |  |  |  | 0.110 ± 0.007  (<0.1–0.12) * | 0.402 ± 0.390  (0.1–1.3) | 8 | W | [13] |
|  |  |  |  |  | 0.283 ± 0.194  (<0.1–0.54) ** | 1.692 ± 1.506  (0.44–5.1) | 8 | D |  |
|  | Crystal River | 0.1 ± 0.01  (0.04–0.1) |  |  |  |  | 9 | W | [1] |
| *Trichechus manatus* | Crystal, River |  |  |  | (<0.02–0.02) | 2.7 ± 0.6  (1.8–4.4) | 19 | D | [14] |

STW = sample type weight; W = wet weight; D = dry weight; *n* = sample size; ^a^ mature animals; ᵇ immature animals; ᶜ more than 17 years-old; ᵈ 39 years-old; * *n* = 2; ** *n* = 6; n. s. = Not specified.

**Table S4.** Toxic metal concentrations (mean ± standard deviation and range; mg/kg; wet weight) in the skin of manatees.

| **Species** | **Location** | **As** | **Cd** | **Cr** | **Hg** | **Pb** | **Reference** |
| --- | --- | --- | --- | --- | --- | --- | --- |
| *Trichechus manatus latirostris* | Crystal River, Florida, USA | 0.050 ± 0.017  (0.029–0.082;  *n* = 8) | 0.036 ± 0.021  (0.005–0.067; *n* = 8) |  | 0.002 ± 0.001  (<L.D.–0.003; *n* = 7) | 0.036 ± 0.058 (0.006–0.178; *n* = 8) | [4] |
|  |  | 0.10 ± 0.01  (0.002–0.3;  *n* = 26) |  |  |  |  | [1] |
| *Trichechus manatus manatus* | Laguna de Términos, Campeche, Mexico. |  | 0.016  (<L.D.–0.032) | 0.783  (0.725–0.841) | <L.D. | 0.265  (<L.D.–0.529) | [15] |

*n* = sample size. L.D. = limit of detection.

**Table S5.** Toxic metal concentrations (mean ± standard deviation and range; mg/kg) in the muscle of dugongs and manatees around the world.

| **Species** | **Location** | **As** | **Cd** | **Cr** | **Hg** | **MeHg** | **Pb** | **STW** | **Reference** |
| --- | --- | --- | --- | --- | --- | --- | --- | --- | --- |
| *Dugong dugon* | Queensland´s coastal, Australia (*n* = 25) |  | <0.1–<0.2 | <0.3–<0.5 |  |  | <0.3–<0.5 | D | [6] |
|  | Cleveland bay, Australia (*n* = 2) |  |  |  | <L. D.–0.01 |  |  | W | [7] |
|  | Northern Australia  (*n* = 1) |  | <0.16 * |  |  |  |  | D | [9] |
|  | Laikang Bay, Sulawesi Island, Indonesia  (*n* = 1) | 0.015 ** | 0.120 ** |  | 0.005 ** | 0.004 ** | 0.25 ** | W | [16] |
|  | (*n* = 1) | 0.050 *** | 0.031 *** |  | 0.002 *** | <0.001 *** | 0.20 *** | W |  |
|  | Torres Strait, Australia  (*n* = 2) ͣ | 0.04 ± 0.01  (0.03–0.05) | 0.015 ± 0.07 (0.01–0.02) |  | <L. D. |  | 0.035 ± 0.007 (0.03–0.04) | W | [11] |
|  | (*n* = 2) ͣ,ᵇ | 3.13 ± 4.33  (0.07–6.2) | 0.02 ± 0.014 |  | <L. D. |  | 0.025 ± 0.007 (0.02–0.03) | W |  |
|  | (*n* = 1) ᵇ | 0.07 | 0.03 |  | <L. D. | . | 0.02 | W | [10] |
| *Trichechus manatus latirostris* | Crystal River, Florida, USA (*n* = 6) | 0.10 ± 0.01  (0.03–0.10) |  |  |  |  |  | W | [1] |
| *Trichechus manatus* | Crystal River, Florida, USA |  |  |  | <0.02 |  |  | D | [14] |

STW = Sample type weight; W = wet weight; D = Dry weight; *n* = sample number; L. D. = Limit of detection; * male more than 17 years-old; ** female (18 years-old, mature); *** female (7 years-old, immature). ^a^ Great Barrier reef; ᵇ muscle plus fat; MeHg = Methyl mercury.

**Table S6.** Toxic metal concentrations (mean ± standard deviation and range; mg/kg) in the kidney of dugongs and manatees around the world.

| **Species** | **Location** | **As** | **Cd** | **Cr** | **Hg** | **Pb** | **STW** | **Reference** |
| --- | --- | --- | --- | --- | --- | --- | --- | --- |
| *Dugong dugon* | Queensland, Australia (*n* = 28) |  | 0.2–309.0 | <0.2–<0.3 |  | <0.1–<0.3 | D | [6] |
|  | Townsville's coastal, Australia (*n* = 2) |  |  |  | <0.01–0.05 |  | W | [7] |
|  | Northern, Australia (*n* = n.s.) |  | 57.0 |  |  |  | D | [9] |
|  | Torres Strait, Australia (*n* = 1) | 0.35 | 17.0 |  | 0.04 | 0.07 | W | [10] |
|  | Torres Strait: Great Barrier reef, Australia (*n* = 3) | 0.02 ± 0.08  (0.19–0.35) | 8.17 ± 7.72  (2.7–17.0) |  | 0.02 ± 0.02 (0.01–0.04) | 0.06 ± 0.01 (0.04–0.07) | W | [11] |
| *Trichechus manatus latirostris* | Caloosahatchee River, Florida, USA (*n* = 8) |  | 1.2–22.4 |  |  |  | W | [13] |
|  | Crystal, River, Florida, USA (*n* = 9) | 0.1 ± 0.01  (0.03–0.1) |  |  |  |  | W | [1] |
| *Trichechus manatus* | Crystal Rival, Florida, USA |  | 25.7 ± 42.6 (<0.1–190.0; *n* = 36) |  |  | 5.2 ± 1.0  (3.3–7.1; *n* = 20) | D | [14] |

STW = Sample type weight; W = wet weight; D = Dry weight; *n* = sample size. n. s. = not specified.

**Table S7.** Toxic metal concentrations (mean ± standard deviation and range; mg/kg) in the brain of dugongs and manatees.

| **Species.** | **Location** | **As** | **Cd** | **Cr** | **Hg** | **Pb** | **Age (Years)** | **STW** | **Reference** |
| --- | --- | --- | --- | --- | --- | --- | --- | --- | --- |
| *Dugong dugon* | Townsville, Australia (*n* = 3) |  | 0.1 | <0.3 |  | <0.5 | 13 | D | [14] |
|  |  |  | 0.1 | <0.3 |  | <0.5 | 24 | W |  |
|  |  |  | 0.2 | <0.3 |  | <0.5 | 31 | W |  |
| *Trichechus manatus latirostris* | Caloosahatchee River, Florida (*n* = 2) |  |  |  | <0.1 | <0.1–0.1 |  | W | [13] |
|  |  |  |  |  | <0.1–0.11 | <0.1–0.5 |  | D |  |
|  | Crystal River, Florida (*n* = 5) | 0.03 ± 0.01  (0.01–0.1) |  |  |  |  |  | W | [1] |
|  | | | | | | | | | |

STW = Sample type weight. W = wet weight; D = Dry weight; *n* = sample size.

**Table S8.** Toxic metal concentrations (mean ± standard deviation and range; mg/kg) or percentage in bone of dugongs and manatees.

| **Species** | **Location** | **Tissue** | **As** | **Cd** | **Cr** | **Hg** | **Pb** | **Unit** | **STW** | **References** |
| --- | --- | --- | --- | --- | --- | --- | --- | --- | --- | --- |
| *Dugong dugon* | PMBC, Thailand  (*n* = 43) | Crown |  | 0.024 ± 0.005 | 0.015 ± 0.026 |  | 0.001 ± 0.001 | % | D | [17] |
|  |  | Root |  | 0.022 ± 0.006 | 0.010 ± 0.003 |  | 0.001 ± 0.001 |  |  |  |
|  |  | Superficial tusk |  | 0.021 ± 0.003 | 0.009 ± 0.002 |  | 0.001 ± 0.001 |  |  |  |
|  |  | Intermediate tusk |  | 0.019 ± 0.002 | 0.008 ± 0.002 |  | <L. D. |  |  |  |
|  |  | Medial tusk |  | 0.020 ± 0.002 | 0.008 ± 0.001 |  | <L. D. |  |  |  |
| *Trichechus manatus manatus* | Quintana Roo, Mexico (*n* = 19) | Bone |  |  |  | 0.1–3.2 | 128.0 | mg/kg | W | [18] |
|  | Mexican Caribbean  (*n* = 22) | Cortical | 0.028 ± 0.026  (<L. D.–0.30) | 3.9 ± 0.5  (3.2–4.9) | 6.8 ± 1.9  (3.1–10.7) |  | 11.2 ± 3.2  (6.0–17.7) |  |  | [19] |
|  | The Gulf of Mexico  (*n* =11) |  | 0.012 ± 0.002  (<L. D.–0.014) | 4.1 ± 0.4  (3.5–4.6) | 9.1 ± 1.1  (6.8–11.2) |  | 14 ± 2.4  (16.4–8.8) |  |  |  |
|  | Chetumal Bay, Mexico | Skull *n* = 4 |  | 3.8 ± 0.45  (3.0–4.0) | 2.9 ± 0.36  (2.6–3.4) | 0.6 ± 0.64  (0.2–1.9) *n* = 5 | 55.6 ± 40  (34–128) |  |  | [20] |
|  |  | Vertebreates *n* = 1 |  | 5.0 | 3.3 | 0.63 | 44.0 |  |  |  |
|  |  | Ribs *n* = 9 |  | 4.6 ± 0.5  (4–5) | 3.34 ± 0.16  (3.1–3.7) | 0.74 ± 0.94  (0.1–3.2) | 41.5 ± 2.7  (37–46) |  |  |  |
|  |  | Flipper *n* = 2 |  | 5 ± 0 | 3.4 ± 0 | 0.45 ± 0.35  (0.2–0.7) | 44 ± 1.4  (43–45) |  |  |  |
|  | Holbox | Vertebreates *n* = 1 |  | 5 | 3.3 | 0.7 | 47 |  |  |  |

STW = sample type weight. PMBC = Phuket Marine Biological Center, Reference Collection. L.D. = Limit of detection.

**Table S9.** Toxic metal concentrations (mean ± standard deviation and range, or unique value reported; mg/kg; wet weight) in intestine and gonads of dugong and manatees.

| **Especie** | **Tissue** | **Location** | **As** | **Cd** | **Hg** | **Pb** | **Reference** |
| --- | --- | --- | --- | --- | --- | --- | --- |
| *Dugong dugon* | Intestine | Torres Strait: Great Barrier reef, Australia (*n* = 1) | 0.08 | 0.09 | L. D. | 0.03 | [11] |
|  | Gonads | Cleveland Bay, Australia (*n* = 2) |  |  | <0.01 |  | [7] |
| *Trichechus manatus latirostris* | Intestine | Florida, USA (*n* = 4) | 0.10 ± 0.03 (0.01–0.2) |  |  |  | [1] |

L.D. = Limit of detection.

**Table S10.** Concentration of mercury (mean ± standard deviation and range, when applicable; mg/kg) in marine mammals around the world.

| **Group** | **Species** | **Blood** | ***n*** | **Muscle** | ***n*** | **Liver** | ***n*** | **Location** | **Reference** | **STW** |
| --- | --- | --- | --- | --- | --- | --- | --- | --- | --- | --- |
| Odontocetes | *Globicephala melas* |  |  | 3.06 ± 2.60 | 20 | 64.9 ± 164 * | 21 | Scotland, United Kingdom | [21] | W |
|  |  |  |  | 2.62 ± 1.10 ^ꝉ^ | 20 | 2.75 ± 2.02 ^ꝉ^ | 21 |  |  |  |
|  | *Balaena mysticetus* |  |  | 0.02  (0.003–0.040) | n. s. | 0.05  (0.01–0.19) | n. s. | Barrow, Alaska, USA | [22] | W |
|  | *Neophocaena phocaenoides* |  |  |  |  | 36.97 *  (1.04–490.23) | 22 | Pearl River  Estuary coast, China | [23] | D |
|  | *Sousa chinensis* |  |  | 0.52 ± 0.44  (0.03–1.84) | 29 | 46.77 ± 72.23  (0.13–216.71) | 10 | South Sea, China | [24] | D |
|  | *Delphinus delphis* |  |  | 0.9 ± 0.08 *  (0.1–1.8) | 36 | 16.7 ± 2.9 *  (0.5–66.0) | 36 | Portugal | [25] | W |
|  | *Tursiops truncates* |  |  | 4.44 ± 1.10 *  (0.52–26.91) | 25 | 131.49 ± 30.31 *  (2.27–524.28) | 25 | Portugal | [26] | W |
|  | *Kogia sima* |  |  |  |  | 6.25 ± 1.86 *  (0.21–17.9) | 12 | South Carolina | [27] | W |
|  | *Hharbour porpoises* |  |  |  |  | 30 ± 51  (1.8–292.0) | 105 | North Sea, France and Belgium | [28] | D |
|  | *Sotalia guianensis* |  |  |  |  | 15.46 ± 20.15  (0.17–58.77) | 11 | Northern coast of Rio de Janeiro, Brazil | [29] | W |
|  | *Stella frontalis* |  |  |  |  | 40.27 ± 19.16  (19.55–57.36) | 3 | Northern coast of Rio de Janeiro, Brazil | [29] | W |
|  | *Tursiops truncates* |  |  |  |  | 42.63 ± 46.51  (9.74–75.51) | 2 | Northern coast of Rio de Janeiro, Brazil | [29] | W |
|  | *Sotalia guianensis* |  |  |  |  | 27.77 | 19 | Southeast, Brazil | [30] | D |
|  | *Tursiops aduncus* |  |  |  |  | 475.78 ± 618.81  (0.28–2110.68) | 59 | South Australia | [31] | W |
|  | *Tursiops truncates* |  |  |  |  | 213.94 ± 241.33  (2.50–771.90) | 10 |  |  |  |
|  | *Delphinus delphis* |  |  |  |  | 31.21 ± 37.11  (0.15–165.28) | 68 |  |  |  |
|  | *Tursiops truncates* | 0.147 ± 0.088 | 74 |  |  |  |  | Charleston, South Carolina | [32] | W |
|  |  | 0.086 ± 0.033 ^ꝉ^ | 16 |  |  |  |  |  |  |  |
|  |  | 0.658 ± 0.519 | 75 |  |  |  |  | Indian River Lagoon, Florida |  |  |
|  |  | 0.265 ± 0.135 ^ꝉ^ | 8 |  |  |  |  |  |  |  |
|  | *Delphinapterus leucas* |  |  |  |  | 15.95 ± 15.17  (0.28–72.48) | 48 | Point Lay, Alaska | [33] | D |
|  | *Dugong dugon* |  |  | (<0.1–0.005) | n. s. | (0.03–0.30) | 21 | Australia/Indonesia | [7,11,16,34] |  |

STW = Sample type weight; W = wet weight; D = dry weight; n. s. = not specified; * Median ± standard error; ^ꝉ^ Methylmercury.

References

1. Takeuchi, N.Y. Trace metal concentrations and the physiological role of zinc in the West Indian Manatee (*Trichechus manatus*). Ph.D. Thesis, University of Florida, Gainesville, FL, USA, 2012.
2. Takeuchi, N.Y.; Walsh, M.T.; Bonde, R.K.; Powell, J.A.; Bass, D.A.; Gaspard, J.C.; Barber, D.S. Baseline reference range for trace metal concentrations in whole blood of wild and managed west Indian manatees (*Trichechus manatus*) in Florida and Belize. *Aquat. Mamm.* **2016**, *42*, 440–453, doi:10.1578/AM.42.4.2016.440.
3. Anzolin, D.G.; Sarkis, J.E.S.; Díaz, E.; Soares, D.G.; Serrano, I.L.; Borges, J.C.G.; Souto, A.S.; Taniguchi, S.; Montone, R.C.; Bainy, A.C.D.; et al. Contaminant concentrations, biochemical and hematological biomarkers in blood of West Indian manatees *Trichechus manatus* from Brazil. *Mar. Pollut. Bull*. **2012**, *64*, 1402–1408, doi:10.1016/j.marpolbul.2012.04.018.
4. Stavros, H.-C.W.; Bonde, R.K.; Fair, P.A. Concentrations of trace elements in blood and skin of Florida manatees (*Trichechus manatus latirostris*). *Mar. Pollut. Bull*. **2008**, *56*, 1215–1233, doi:10.1016/j.marpolbul.2008.04.
5. Siegal-Willott, J.L.; Harr, K.E.; Hall, J.O.; Hayek, L.-A.C.; Auil-Gomez, N.; Powell, J.A.; Bonde, R.K.; Heard, D. Blood mineral concentrations in manatees (*Trichechus manatus latirostris* and *Trichechus manatus manatus*). *J. Zoo Wildl. Med*. **2013**, *44*, 285–294, doi:10.1638/2012-0093R.1.
6. Denton, G.R.W.; Marsh, H.; Heinsohn, G.E.; Burdon-Jones, C. The unusual metal status of the Dugong (*Dugong dugon*). *Mar. Biol.* **1980**, *57*, 201–219, doi:10.1007/BF00390738.
7. Denton, G.R.; Breck, W.G. Mercury in tropical marine organisms from North Queensland. *Mar. Pollut. Bull*. **1981**, *12*, 116–121, doi:10.1016/0025-326X(81)90439-2.
8. Parry, D.L. Munksgaard, N.C. Heavy metal baseline data for sediment, seawater, and biota, Bing Bong, Gulf of Carpentaria. Northern Territory University, Darwin, 1992. Quoted in Haynes, D.; Carter, S.; Gaus, C.; Muller J.; Dennison, W. Organochlorine and heavy metal concentrations in blubber and liver tissue collected from Queensland (Australia) dugong (*Dugong dugon*). *Mar. Pollut. Bull*. **2005**, *51*, 361–369, doi:10.1016/j.marpolbul.2004.10.020.
9. Marsh, H. Mass stranding of dugongs by a tropical cyclone in northern Australia. *Mar. Mamm. Sci.* **1989**, *5*, 78–84, doi:10.1111/j.1748-7692.1989.tb00215.x.
10. Dight, I.; Gladstone, W. *Torres Strait Baseline Study: Pilot Study Final Report June 1993, Research Publication 29*; Great Barrier Reef Marine Park Authority: Townsville Qld, Australia, 1993; pp. 1–259, ISBN 0642 173B7 7.
11. Gladstone, W. *Trace Metals in Sediments, Indicator Organisms and Traditional Seafoods of the Torres Strait, Report Series 5a*; Great Barrier Reef Marine Park Authority: Queensland, Australia, 1996; ISBN 0 642 25478 8.
12. Haynes, D.; Kwan, D.; Trace Metal Concentrations in the Torres Strait Environment and Traditional Seafood Species, 1997–2000; Torres Strait Regional Authority, Thursday Island: Queensland, Australia, 2001. Quoted in Haynes, D.; Carter, S.; Gaus, C.; Muller J.; Dennison, W. Organochlorine and heavy metal concentrations in blubber and liver tissue collected from Queensland (Australia) dugong (*Dugong dugon*). *Mar. Pollut. Bull*. **2005**, *51*, 361–369, doi:10.1016/j.marpolbul.2004.10.020.
13. O’Shea, T.J.; Rathbun, G.B.; Bonde, R.K. An epizootic of Florida manatees associated with a dinoflagellate bloom. *Mar. Mamm. Sci.* **1991**, *7*, 165–179, doi:10.1111/j.1748-7692.1991.tb00563.x.
14. O’Shea, T.J.; Moore, J.F.; Kochman, H.I. Contaminant concentrations in manatees in Florida. *J. Wildl. Manag.* **1984**, *3*, 741–748, doi:10.2307/3801421.
15. Benítez, J.A.; Vidal, J.; Brichieri-Colombi, T.; Delgado-Estrella, A. Monitoring ecosystem health of the Terminos Lagoon region using heavy metals as environmental indicators. *Environ. Impact* **2012**, *162*, 349–358, doi:10.2495/EID120311.
16. Miyazaki, N.; Itano, K.; Fukushima, M.; Kawai, S.-I.; Honda, K. Metals and organochlorine compounds in the muscle of dugong from Sulawesi Island. *Sci. Rep. Whales Res. Inst*. **1979**, *31*, 125–128.
17. Nganvongpanit, K.; Buddhachat, K.; Piboon P.; Euppayo, T.; Kaewmong, P.; Cherdsukjai, P.; Kittiwatanawong, K.; Thitaram, C. Elemental classification of the tusks of dugong (*Dugong dugong*) by HHXRF analysis and comparison with other species. *Sci. Rep.* **2017**, *7*, 1–12, doi:10.1038/srep46167.
18. Rojas-Mingüer, A.; Morales-Vela, B.M.; Rosiles-Martínez, R*. Metals in Bone and Blood of manatees (Trichechus manatus manatus) from Chetumal Bay, Quintana Roo, México. ECOSUR, México*; Vos, J., Bossart G., Fournier, M., Eds.; Taylos & Francis: London, UK, New York, NY, USA, 1997; p. 287.
19. Romero-Calderon, A.G.; Morales-Vela, B.; Rosíles-Martínez, R.; Olivera-Gómez, L.D.; Delgado-Estrella, A. Metals in bone tissue of Antillean Manatees from the Gulf of Mexico and Chetumal Bay, Mexico. *Bull. Environ. Contam. Toxicol.* **2016**, *96*, 9–14, doi:10.1007/s00128-015-1674-6.
20. Rojas-Mingüer, A.; Morales-Vela, B. Metales pesados en hueso y sangre de manatíes (*Trichechus manatus manatus*) de la Bahía de Chetumal, Quintana Roo, México. In *Contribuciones de la Ciencia al Manejo Costero Integrado de la Bahía de Chetumal y su área de influencia*, 2nd ed.; Rosado-May, F.J., Romero-May, R., Navarrete, A., Eds.; Universidad de Quintana Roo: Chetumal, México, 2002; pp. 133–142; ISBN 968-7864-34-6.
21. Gajdosechova, Z.; Brownlow, A.; Cottin, N.T.; Fernandes, M.; Read, F.L.; Urgast D.S.; Raab, A.; Feldmann, J.; Krupp, E.M. Possible link between Hg and Cd accumulation in the brain of long-finned pilot whales (*Globicephala melas*). *Sci. Total Environ.* **2016**, *545*, 407–413, doi:10.1016/j.scitotenv.2015.12.082.
22. O’Hara, T.M.; Woshner, V.; Bratton, G. Inorganic pollutants in Artic marine mammals. In *Toxicology of Marine Mammals*, Vos, J.G., Bossart, G.D., Fournier, M., O`Shea, T.J., Eds.; Taylos & Francis New York, USA, 2003; Volume 3, pp. 206–246; ISBN 0-203-16557-8.
23. Zhang, X.; Lin, W.; Yu, R.-Q.; Sun, X.; Ding, Y.; Chen, H.; Chen, X.; Wu, Y. Tissue partition and risk assessments of trace elements in Indo-Pacific Finless Porpoises (*Neophocaena phocaenoides*) from the Pearl River Estuary coast, China. *Chemosphere* **2017**, *185*, 1197–1207, doi:10.1016/j.chemosphere.2017.07.080.
24. Sun, X.; Yu, R.-Q.; Zhang, M.; Zhang, X.; Chen, X.; Xiao Y.; Ding, Y.; Wu, Y. Correlation of trace element concentrations between epidermis and internal organ tissues in Indo-Pacific humpback dolphins (*Sousa chinensis*). *Sci. Total Environ.* **2017**, *605*, 238–245, doi:10.1016/j.scitotenv.2017.06.180.
25. Monteiro, S.S.; Pereira, A.T.; Costa, E.; Torres, J.; Oliveira, I.; Bastos-Santos, J.; Araújo, H.; Ferreira, M.; Vingada, J.; Eira, C. Bioaccumulation of trace element concentrations in common dolphins (*Delphinus delphis*) from Portugal. *Mar. Pollut. Bull.* **2016**, *113*, 400–407, doi:10.1016/j.marpolbul.2016.10.033.
26. Monteiro, S.S.; Torres, J.; Ferreira, M.; Marçalo, A.; Nicolau, L.; Vingada J.; Eira C. Ecological variables influencing trace element concentrations in bottlenose dolphins (*Tursiops truncatus*, Montagu 1821) stranded in continental Portugal. *Sci. Total Environ.* **2016**, *544*, 837–844, doi:10.1016/j.scitotenv.2015.12.037.
27. Reed, L.A.; McFeeW, E.; Pennington, P.L.; Wirth, E.F.; Fulton, M.H. A survey of trace element distribution in tissues of the dwarf spermwhale (*Kogia sima*) stranded along the South Carolina coast from 1990–2001. *Mar. Pollut. Bull.* **2015**, *100*, 501–506, doi:10.1016/j.marpolbul.2015.09.005.
28. Mahfouz, C.; Henry, F.; Courcot, L.; Pezeril, S.; Bouveroux, T.; Dabin, W.; Jauniaux, T.; Khalaf, G.; Amara, R. Harbour porpoises (*Phocoena phocoena*) stranded along the southern North Sea: An assessment through metallic contamination. *Environ. Res*. **2014**, *133*, 266–273, doi:10.1016/j.envres.2014.06.006.
29. Lemos, L.S.; De Moura, J.F.; Hauser-Davis, R.A.; De Campos, R.C.; Siciliano, S. Small cetaceans found stranded or accidentally captured in southeastern Brazil: Bioindicators of essential and non-essential trace elements in the environment. *Ecotoxicol. Environ. Saf*. **2013**, *97*, 166–175, doi:10.1016/j.ecoenv.2013.07.025.
30. Seixas, T.G.; Kehrig, H.A.; Beneditto, A.P.M.; Souza, C.M.M.; Malm, O.; Moreira, I. Essential (Se, Cu) and non-essential (Ag, Hg, Cd) elements: What are their relationships in liver of *Sotalia guianensis* (Cetacea, Delphinidae)? *Mar. Pollut. Bull.* **2009**, *58*, 601–634, doi:10.1016/j.marpolbul.2008.12.005.
31. Lavery, T.J.; Butterfield, N.; Kemper, C.M.; Reid, J.R.; Sanderson, K. Metals and selenium in the liver and bone of three dolphin species from South Australia, 1988–2004. *Sci. Total Environ.* **2008**, *390*, 77–85, doi:10.1016/j.scitotenv.2007.09.016.
32. Stavros, H.-C.W.; Bossart, G.D.; Hulsey, T.C.; Fair, P.A.; Trace element concentrations in blood of free-ranging bottlenose dolphins (*Tursiops truncatus*): Influence of age, sex and location. *Mar. Pollut. Bull*. **2008**, *56*, 348–379, doi:10.1016/j.marpolbul.2007.10.030.
33. Dehn, L.A.; Follmann, E.H.; Thomas, D.L.; Sheffield, G.G.; Rosa, C.; Duffy, L.K.; O’Hara, T.M. Trophic relationships in an Arctic food web and implications for trace metal transfer. *Sci. Total Environ.* **2006**, *362*, 103–123, doi:10.1016/j.scitotenv.2005.11.012.
34. Haynes, D.; Carter, S.; Gaus, C.; Muller, J.; Dennison, W. Organochlorine and heavy metal concentrations in blubber and liver tissue collected from Queensland (Australia) dugong (*Dugong dugon*). *Mar. Pollut. Bull*. **2005**, *51*, 361–369, doi:10.1016/j.marpolbul.2004.10.020.
