# Supplementary material for: As, Cr, Hg, Pb, and Cd Concentrations and Bioaccumulation in the Dugong Dugong dugon and Manatee Trichechus manatus: A Review of Body Burdens and Distribution
Source: Int J Environ Res Public Health. 2019 Jan 31;16(3):404. doi: 10.3390/ijerph16030404 (PMC6388294; doi:10.3390/ijerph16030404)
Supplement: Supplementary file 1 [file ijerph-16-00404-s001.zip › ijerph-389570-suppl xml-1.pdf]

Table S1. Arsenic concentration (mean ± standard error and range, mg/kg; wet weight) in different body tissues and contents from *Trichechus manatus manatus* carcasses, opportunistic sampling; *n* = sample size (modified from Takeuich [1]).

Tissue	Concentration	*n*	Tissue	Concentration	*n*	Contents	Concentration	*n*
Thyroid	0.3 ± 0.1 (0.01–0.7)	5	Stomach	0.04 ± 0.004 (0.03–0.04)	2	Milk	0.02 ± 0.002 (0.01–0.02)	4
Thymus	0.1 ± 0.02 (0.01–0.1)	7	Duodenum	0.1 ± 0.03 (0.01–0.2)	9	Stomach contents	0.03 ± 0.02 (0.01–0.1)	2
Lung	0.1 ± 0.01 (0.01–0.1)	9	Duodenum horns	0.01	1	Duodenum contents	0.01	1
Cardiac gland	0.01 ± 0.01 (0.002–0.03)	2	Cecum	0.1 ± 0.01 (0.03–0.1)	6	Cecal contents	0.03 ± 0.02 (0.02–0.10)	2
Heart	0.04 ± 0.01 (0.02–0.1)	6	Cecal horns	0.10 ± 0.02 (0.002–0.10)	7	Faeces	0.8 ± 0.1 (0.1–3.4)	23
Spleen	0.05 ± 0.02 (0.03–0.1)	4	Colon	0.04 ± 0.02 (0.01–0.10)	3	Urine	0.2 ± 0.1 (0.0–0.8)	19
Gall bladder	0.1± 0.1 (0.01–0.1)	2	Ovary	0.10 ± 0.03 (0.02–0.10)	2			
Pancreas	0.03 ± 0.01 (0.01–0.1)	7	Uterus	0.04	1			
Blubber	0.1 ± 0.02 (0.02–0.2)	7	Seminal vesicles	0.01	1			
Bladder	0.10 ± 0.04 (0.01–0.10)	2	Testes	0.02	1			

Table S2. Toxic metal concentrations (mean ± standard deviation; µg/kg, wet weight; *n* = sample size) in the blood fraction of manatees *Trichechus manatus*.

Fraction	Species	As	*n*	Cd	*n*	Cr	*n*	Hg	*n*	Pb	*n*	Location	Reference
Whole blood	*T. manatus*	300.0 ± 20.0	77	2.0 ± 0.2	75	11.0 ± 1.0	29	<0.6 ± 0.1	20	30.0 ± 10.0	20	CCF	[2]
		170.0 ± 20.0	20	3.0 ± 0.5	20	36.0 ± 2.0	10	9.0 ± 1.0	20	190.0 ± 2.0	14	BCF	
		190.0 ± 80.0	7	0.4 ± 0.1	7			1.5 ± 0.5	7	31.0 ± 9.0	7	CF	
		200.0 ± 20.0	14	1.0 ± 0.1	14			1.0 ± 0.3	13	9.0 ± 1.0	14	CHF	
		430.0 ± 70.0	33	4.0 ± 0.3	33	<100.0	33		33	<50.0	33	BE	
		63.0 ± 11.0	24	3.4 ± 0.4	24	<100.0	24		24	<50.0	14	BE *	
				1.7 ± 1.1	8	10.0 ± 3.0	8			50.0 ± 20.0	8	PB	[3]
				1.9 ± 1.0	4	7.0 ± 3.0	4			43.0 ± 7.0	4	AB	
				8.2 ± 14.5	4	9.0 ± 2.0	4			100.0 ± 66.0	4	PAB	
	T. manatus latirostris	342.0 ± 44.0	8	1.0 ± 1.0	8			8.0 ± 17.0	7	13.0 ± 3.0	8	CRF	[4]
	T. manatus + *T. manatus latirostris*	493.0 ± 250.0	45	1.0 ± 0.0	45	820.0 ± 80.0	45	1.01 ± 1.00	45	52.0 ± 110.0	45	B/F	[5]
Plasma	*T. manatus*	30.0 ± 10.0	40	100 ± 50	39	10.0 ± 1.0	31					CCF	[1]
		10.0 ± 2.0	10	<10.0	10	0.1 ± 0.1	3					BCF	
		10.0 ± 2.0	16	<10.0	16	20.0 ± 10.0	16					BE	
		10.0 ± 2.0	8	<10.0	8	10.0 ± 3.0	8					F *	
Erythrocytes	*T. manatus*	500.0 ± 40.0	67	2.0 ± 0.1	64	10.0 ± 2.0	51	20.0 ± 4.0	64	40.0 ± 10.0	51	CCF	
		300.0 ± 30.0	11	0.1 ± 0.1	11	4.0 ± 0.4.0	3			4.0 ± 4.0	3	BCF	
		400.0 ± 100.0	10	1.0 ± 0.1	7	10.0 ± 9.0	8	3.0 ± 1.0	8	100.0 ± 20.0	8	CCF	
		300.0 ± 30.0	9	1.0	9	1.0 ± 0.3	9	2.0 ± 0.2	9	30.0 ± 5.0	9	CCF	
		1300.0 ± 300.0	15	<10.0 ± 0.02	15	100.0 ± 30.0	15	<10.0 ± 0.004	15	30.0 ± 10.0	15	BE	
		100.0 ± 20.0	9	<10.0 ± 10.0	9	70.0 ± 20.0	9			20.0 ± 4.0	9	F *	
Serum	*T. manatus* + *T. manatus latirostris*	17.0 ± 20.0	45	1.0 ± 0.0	45	386.0 ± 30.0	45	1.0 ± 0.0	45	3.0 ± 5.0	45	B/F	[5]

* Cautivity. CCF = Citrus County, Florida: USA; BCF = Brevard County, Florida: USA; CF = Collier Florida: USA; CHF = Charllot Florida: USA; BE = Belize; B/F = Belize and Florida; PB = Pernambuco, Brazil; AB = Alagoas, Brazil: PAB = Parabai, Brazil; CRF = Crystal River, Florida: USA; F = Florida.

Table S3. Toxic metal concentrations (mean ± standard deviation and range; mg/kg) in liver of dugongs and manatees from Australia and Florida.

Species	Location	As	Cd	Cr	Hg	Pb	*n*	STW	Reference
Dugong dugon (Australia)	Queensland coast	3.0 ± 1.9 (0.45–7.7)	(<0.005–32.5)	2.7 ± 4.0 (0.2–18)	0.3 ± 0.29 (0.05–1.11)	(<0.08–3.08)	18 [a]	W	[6]
		2.1 ± 1.6 (0.04–5.3)	(<0.005–3.0)	(<0.2–10.2)	0.09 ± 0.06 (0.04–0.28)	(<0.08–0.85)	20 [b]		
			(<0.1–58.8)	(<0.2–<0.5)		(<0.1–<0.3)	42	D	[6]
	Northern Territory				0.05		2	W	[7]
	McArthur River	(0.2–0.8)	(10.0–15.0)		(<0.02–0.03)	(0.17–0.42)	n. s.	D	[8]
	Northern, Territory		16.4				1 [c]	D	[9]
			36.6				1 [d]	D	
	Torres Strait	0.23	4.90		0.04	0.05	1	W	[10]
	Torres Strait: Great Barrier reef	0.27 ± 0.11 (0.18–0.40)	6.43 ± 2.74 (4.8–9.6)		0.03 ± 0.01 (0.02–0.04)	0.08 ± 0.029 (0.05–0.10)	3	W	[11]
		(0.26–2.00)	(0.44–54.00)	(<0.1–2.9)	(<0.005–0.22)	<0.04	36	D	[12]
Trichechus manatus latirostris (Florida)	Caloosahatchee River				0.110 ± 0.007 (<0.1–0.12) *	0.402 ± 0.390 (0.1–1.3)	8	W	[13]
					0.283 ± 0.194 (<0.1–0.54) **	1.692 ± 1.506 (0.44–5.1)	8	D	
	Crystal River	0.1 ± 0.01 (0.04–0.1)					9	W	[1]
Trichechus manatus	Crystal, River				(<0.02–0.02)	2.7 ± 0.6 (1.8–4.4)	19	D	[14]

STW = sample type weight; W = wet weight; D = dry weight; *n* = sample size; [a] mature animals; [b] immature animals; [c] more than 17 years-old; [d] 39 years-old; * *n* = 2; ** *n* = 6; n. s. = Not specified.

Table S4. Toxic metal concentrations (mean ± standard deviation and range; mg/kg; wet weight) in the skin of manatees.

Species	Location	As	Cd	Cr	Hg	Pb	Reference
Trichechus manatus latirostris	Crystal River, Florida, USA	0.050 ± 0.017 (0.029–0.082; *n* = 8)	0.036 ± 0.021 (0.005–0.067; *n* = 8)		0.002 ± 0.001 (<L.D.–0.003; *n* = 7)	0.036 ± 0.058 (0.006–0.178; *n* = 8)	[4]
		0.10 ± 0.01 (0.002–0.3; *n* = 26)					[1]
Trichechus manatus manatus	Laguna de Términos, Campeche, Mexico.		0.016 (<L.D.–0.032)	0.783 (0.725–0.841)	<L.D.	0.265 (<L.D.–0.529)	[15]

n = sample size. L.D. = limit of detection.

Table S5. Toxic metal concentrations (mean ± standard deviation and range; mg/kg) in the muscle of dugongs and manatees around the world.

Species	Location	As	Cd	Cr	Hg	MeHg	Pb	STW	Reference
Dugong dugon	Queensland´s coastal, Australia (*n* = 25)		<0.1–<0.2	<0.3–<0.5			<0.3–<0.5	D	[6]
	Cleveland bay, Australia (*n* = 2)				<L. D.–0.01			W	[7]
	Northern Australia (*n* = 1)		<0.16 *					D	[9]
	Laikang Bay, Sulawesi Island, Indonesia (*n* = 1)	0.015 **	0.120 **		0.005 **	0.004 **	0.25 **	W	[16]
	(*n* = 1)	0.050 ***	0.031 ***		0.002 ***	<0.001 ***	0.20 ***	W	
	Torres Strait, Australia (*n* = 2) [a]	0.04 ± 0.01 (0.03–0.05)	0.015 ± 0.07 (0.01–0.02)		<L. D.		0.035 ± 0.007 (0.03–0.04)	W	[11]
	(*n* = 2) [a,b]	3.13 ± 4.33 (0.07–6.2)	0.02 ± 0.014		<L. D.		0.025 ± 0.007 (0.02–0.03)	W	
	(*n* = 1) [b]	0.07	0.03		<L. D.	.	0.02	W	[10]
Trichechus manatus latirostris	Crystal River, Florida, USA (*n* = 6)	0.10 ± 0.01 (0.03–0.10)						W	[1]
Trichechus manatus	Crystal River, Florida, USA				<0.02			D	[14]

STW = Sample type weight; W = wet weight; D = Dry weight; *n* = sample number; L. D. = Limit of detection; * male more than 17 years-old; ** female (18 years-old, mature); *** female (7 years-old, immature). [a] Great Barrier reef; [b] muscle plus fat; MeHg = Methyl mercury.

Table S6. Toxic metal concentrations (mean ± standard deviation and range; mg/kg) in the kidney of dugongs and manatees around the world.

Species	Location	As	Cd	Cr	Hg	Pb	STW	Reference
Dugong dugon	Queensland, Australia (*n* = 28)		0.2–309.0	<0.2–<0.3		<0.1–<0.3	D	[6]
	Townsville's coastal, Australia (*n* = 2)				<0.01–0.05		W	[7]
	Northern, Australia (*n* = n.s.)		57.0				D	[9]
	Torres Strait, Australia (*n* = 1)	0.35	17.0		0.04	0.07	W	[10]
	Torres Strait: Great Barrier reef, Australia (*n* = 3)	0.02 ± 0.08 (0.19–0.35)	8.17 ± 7.72 (2.7–17.0)		0.02 ± 0.02 (0.01–0.04)	0.06 ± 0.01 (0.04–0.07)	W	[11]
Trichechus manatus latirostris	Caloosahatchee River, Florida, USA (*n* = 8)		1.2–22.4				W	[13]
	Crystal, River, Florida, USA (*n* = 9)	0.1 ± 0.01 (0.03–0.1)					W	[1]
Trichechus manatus	Crystal Rival, Florida, USA		25.7 ± 42.6 (<0.1–190.0; *n* = 36)			5.2 ± 1.0 (3.3–7.1; *n* = 20)	D	[14]

STW = Sample type weight; W = wet weight; D = Dry weight; *n* = sample size. n. s. = not specified.

Table S7. Toxic metal concentrations (mean ± standard deviation and range; mg/kg) in the brain of dugongs and manatees.

Species.	Location	As	Cd	Cr	Hg	Pb	Age (Years)	STW	Reference
Dugong dugon	Townsville, Australia (*n* = 3)		0.1	<0.3		<0.5	13	D	[14]
			0.1	<0.3		<0.5	24	W	
			0.2	<0.3		<0.5	31	W	
Trichechus manatus latirostris	Caloosahatchee River, Florida (*n* = 2)				<0.1	<0.1–0.1		W	[13]
					<0.1–0.11	<0.1–0.5		D	
	Crystal River, Florida (*n* = 5)	0.03 ± 0.01 (0.01–0.1)						W	[1]

STW = Sample type weight. W = wet weight; D = Dry weight; *n* = sample size.

Table S8. Toxic metal concentrations (mean ± standard deviation and range; mg/kg) or percentage in bone of dugongs and manatees.

Species	Location	Tissue	As	Cd	Cr	Hg	Pb	Unit	STW	References
Dugong dugon	PMBC, Thailand (*n* = 43)	Crown		0.024 ± 0.005	0.015 ± 0.026		0.001 ± 0.001	%	D	[17]
		Root		0.022 ± 0.006	0.010 ± 0.003		0.001 ± 0.001			
		Superficial tusk		0.021 ± 0.003	0.009 ± 0.002		0.001 ± 0.001			
		Intermediate tusk		0.019 ± 0.002	0.008 ± 0.002		<L. D.			
		Medial tusk		0.020 ± 0.002	0.008 ± 0.001		<L. D.			
Trichechus manatus manatus	Quintana Roo, Mexico (*n* = 19)	Bone				0.1–3.2	128.0	mg/kg	W	[18]
	Mexican Caribbean (*n* = 22)	Cortical	0.028 ± 0.026 (<L. D.–0.30)	3.9 ± 0.5 (3.2–4.9)	6.8 ± 1.9 (3.1–10.7)		11.2 ± 3.2 (6.0–17.7)			[19]
	The Gulf of Mexico (*n* =11)		0.012 ± 0.002 (<L. D.–0.014)	4.1 ± 0.4 (3.5–4.6)	9.1 ± 1.1 (6.8–11.2)		14 ± 2.4 (16.4–8.8)			
	Chetumal Bay, Mexico	Skull *n* = 4		3.8 ± 0.45 (3.0–4.0)	2.9 ± 0.36 (2.6–3.4)	0.6 ± 0.64 (0.2–1.9) *n* = 5	55.6 ± 40 (34–128)			[20]
		Vertebreates *n* = 1		5.0	3.3	0.63	44.0			
		Ribs *n* = 9		4.6 ± 0.5 (4–5)	3.34 ± 0.16 (3.1–3.7)	0.74 ± 0.94 (0.1–3.2)	41.5 ± 2.7 (37–46)			
		Flipper *n* = 2		5 ± 0	3.4 ± 0	0.45 ± 0.35 (0.2–0.7)	44 ± 1.4 (43–45)			
	Holbox	Vertebreates *n* = 1		5	3.3	0.7	47			

STW = sample type weight. PMBC = Phuket Marine Biological Center, Reference Collection. L.D. = Limit of detection.

Table S9. Toxic metal concentrations (mean ± standard deviation and range, or unique value reported; mg/kg; wet weight) in intestine and gonads of dugong and manatees.

Especie	Tissue	Location	As	Cd	Hg	Pb	Reference
Dugong dugon	Intestine	Torres Strait: Great Barrier reef, Australia (*n* = 1)	0.08	0.09	L. D.	0.03	[11]
	Gonads	Cleveland Bay, Australia (*n* = 2)			<0.01		[7]
Trichechus manatus latirostris	Intestine	Florida, USA (*n* = 4)	0.10 ± 0.03 (0.01–0.2)				[1]

L.D. = Limit of detection.

Table S10. Concentration of mercury (mean ± standard deviation and range, when applicable; mg/kg) in marine mammals around the world.

Group	Species	Blood	*n*	Muscle	*n*	Liver	*n*	Location	Reference	STW
Odontocetes	*Globicephala melas*			3.06 ± 2.60	20	64.9 ± 164 *	21	Scotland, United Kingdom	[21]	W
				2.62 ± 1.10 ◦	20	2.75 ± 2.02 ◦	21			
	Balaena mysticetus			0.02 (0.003–0.040)	n. s.	0.05 (0.01–0.19)	n. s.	Barrow, Alaska, USA	[22]	W
	Neophocaena phocaenoides					36.97 * (1.04–490.23)	22	Pearl River Estuary coast, China	[23]	D
	Sousa chinensis			0.52 ± 0.44 (0.03–1.84)	29	46.77 ± 72.23 (0.13–216.71)	10	South Sea, China	[24]	D
	Delphinus delphis			0.9 ± 0.08 * (0.1–1.8)	36	16.7 ± 2.9 * (0.5–66.0)	36	Portugal	[25]	W
	Tursiops truncates			4.44 ± 1.10 * (0.52–26.91)	25	131.49 ± 30.31 * (2.27–524.28)	25	Portugal	[26]	W
	Kogia sima					6.25 ± 1.86 * (0.21–17.9)	12	South Carolina	[27]	W
	Hharbour porpoises					30 ± 51 (1.8–292.0)	105	North Sea, France and Belgium	[28]	D
	Sotalia guianensis					15.46 ± 20.15 (0.17–58.77)	11	Northern coast of Rio de Janeiro, Brazil	[29]	W
	Stella frontalis					40.27 ± 19.16 (19.55–57.36)	3	Northern coast of Rio de Janeiro, Brazil	[29]	W
	Tursiops truncates					42.63 ± 46.51 (9.74–75.51)	2	Northern coast of Rio de Janeiro, Brazil	[29]	W
	Sotalia guianensis					27.77	19	Southeast, Brazil	[30]	D
	Tursiops aduncus					475.78 ± 618.81 (0.28–2110.68)	59	South Australia	[31]	W
	Tursiops truncates					213.94 ± 241.33 (2.50–771.90)	10			
	Delphinus delphis					31.21 ± 37.11 (0.15–165.28)	68			
	Tursiops truncates	0.147 ± 0.088	74					Charleston, South Carolina	[32]	W
		0.086 ± 0.033 ◦	16							
		0.658 ± 0.519	75					Indian River Lagoon, Florida		
		0.265 ± 0.135 ◦	8							
	Delphinapterus leucas					15.95 ± 15.17 (0.28–72.48)	48	Point Lay, Alaska	[33]	D
	Dugong dugon			(<0.1–0.005)	n. s.	(0.03–0.30)	21	Australia/Indonesia	[7,11,16,34]	

STW = Sample type weight; W = wet weight; D = dry weight; n. s. = not specified; * Median ± standard error; ◦ Methylmercury.

References

1. Takeuchi, N.Y. Trace metal concentrations and the physiological role of zinc in the West Indian Manatee (*Trichechus manatus*). Ph.D. Thesis, University of Florida, Gainesville, FL, USA, 2012.
2. Takeuchi, N.Y.; Walsh, M.T.; Bonde, R.K.; Powell, J.A.; Bass, D.A.; Gaspard, J.C.; Barber, D.S. Baseline reference range for trace metal concentrations in whole blood of wild and managed west Indian manatees (*Trichechus manatus*) in Florida and Belize. *Aquat. Mamm.* **2016**, *42*, 440–453, doi:10.1578/AM.42.4.2016.440.
3. Anzolin, D.G.; Sarkis, J.E.S.; Díaz, E.; Soares, D.G.; Serrano, I.L.; Borges, J.C.G.; Souto, A.S.; Taniguchi, S.; Montone, R.C.; Bainy, A.C.D.; et al. Contaminant concentrations, biochemical and hematological biomarkers in blood of West Indian manatees *Trichechus manatus* from Brazil. *Mar. Pollut. Bull*. **2012**, *64*, 1402–1408, doi:10.1016/j.marpolbul.2012.04.018.
4. Stavros, H.-C.W.; Bonde, R.K.; Fair, P.A. Concentrations of trace elements in blood and skin of Florida manatees (*Trichechus manatus latirostris*). *Mar. Pollut. Bull*. **2008**, *56*, 1215–1233, doi:10.1016/j.marpolbul.2008.04.
5. Siegal-Willott, J.L.; Harr, K.E.; Hall, J.O.; Hayek, L.-A.C.; Auil-Gomez, N.; Powell, J.A.; Bonde, R.K.; Heard, D. Blood mineral concentrations in manatees (*Trichechus manatus latirostris* and *Trichechus manatus manatus*). *J. Zoo Wildl. Med*. **2013**, *44*, 285–294, doi:10.1638/2012-0093R.1.
6. Denton, G.R.W.; Marsh, H.; Heinsohn, G.E.; Burdon-Jones, C. The unusual metal status of the Dugong (*Dugong dugon*). *Mar. Biol.* **1980**, *57*, 201–219, doi:10.1007/BF00390738.
7. Denton, G.R.; Breck, W.G. Mercury in tropical marine organisms from North Queensland. *Mar. Pollut. Bull*. **1981**, *12*, 116–121, doi:10.1016/0025-326X(81)90439-2.
8. Parry, D.L. Munksgaard, N.C. Heavy metal baseline data for sediment, seawater, and biota, Bing Bong, Gulf of Carpentaria. Northern Territory University, Darwin, 1992. Quoted in Haynes, D.; Carter, S.; Gaus, C.; Muller J.; Dennison, W. Organochlorine and heavy metal concentrations in blubber and liver tissue collected from Queensland (Australia) dugong (*Dugong dugon*). *Mar. Pollut. Bull*. **2005**, *51*, 361–369, doi:10.1016/j.marpolbul.2004.10.020.
9. Marsh, H. Mass stranding of dugongs by a tropical cyclone in northern Australia. *Mar. Mamm. Sci.* **1989**, *5*, 78–84, doi:10.1111/j.1748-7692.1989.tb00215.x.
10. Dight, I.; Gladstone, W. *Torres Strait Baseline Study: Pilot Study Final Report June 1993, Research Publication 29*; Great Barrier Reef Marine Park Authority: Townsville Qld, Australia, 1993; pp. 1–259, ISBN 0642 173B7 7.
11. Gladstone, W. *Trace Metals in Sediments, Indicator Organisms and Traditional Seafoods of the Torres Strait, Report Series 5a*; Great Barrier Reef Marine Park Authority: Queensland, Australia, 1996; ISBN 0 642 25478 8.
12. Haynes, D.; Kwan, D.; Trace Metal Concentrations in the Torres Strait Environment and Traditional Seafood Species, 1997–2000; Torres Strait Regional Authority, Thursday Island: Queensland, Australia, 2001. Quoted in Haynes, D.; Carter, S.; Gaus, C.; Muller J.; Dennison, W. Organochlorine and heavy metal concentrations in blubber and liver tissue collected from Queensland (Australia) dugong (*Dugong dugon*). *Mar. Pollut. Bull*. **2005**, *51*, 361–369, doi:10.1016/j.marpolbul.2004.10.020.

13. O'Shea, T.J.; Rathbun, G.B.; Bonde, R.K. An epizootic of Florida manatees associated with a dinoflagellate bloom. *Mar. Mamm. Sci.* **1991**, *7*, 165–179, doi:10.1111/j.1748-7692.1991.tb00563.x.
14. O'Shea, T.J.; Moore, J.F.; Kochman, H.I. Contaminant concentrations in manatees in Florida. *J. Wildl. Manag.* **1984**, *3*, 741–748, doi:10.2307/3801421.
15. Benítez, J.A.; Vidal, J.; Brichieri-Colombi, T.; Delgado-Estrella, A. Monitoring ecosystem health of the Terminos Lagoon region using heavy metals as environmental indicators. *Environ. Impact* **2012**, *162*, 349–358, doi:10.2495/EID120311.
16. Miyazaki, N.; Itano, K.; Fukushima, M.; Kawai, S.-I.; Honda, K. Metals and organochlorine compounds in the muscle of dugong from Sulawesi Island. *Sci. Rep. Whales Res. Inst.* **1979**, *31*, 125–128.
17. Nganvongpanit, K.; Buddhachat, K.; Piboon P.; Euppayo, T.; Kaewmong, P.; Cherdsukjai, P.; Kittiwatanawong, K.; Thitaram, C. Elemental classification of the tusks of dugong (*Dugong dugong*) by HHXRF analysis and comparison with other species. *Sci. Rep.* **2017**, *7*, 1–12, doi:10.1038/srep46167.
18. Rojas-Mingüer, A.; Morales-Vela, B.M.; Rosiles-Martínez, R. *Metals in Bone and Blood of manatees (Trichechus manatus manatus) from Chetumal Bay, Quintana Roo, México. ECOSUR, México*; Vos, J., Bossart G., Fournier, M., Eds.; Taylos & Francis: London, UK, New York, NY, USA, 1997; p. 287.
19. Romero-Calderon, A.G.; Morales-Vela, B.; Rosíles-Martínez, R.; Olivera-Gómez, L.D.; Delgado-Estrella, A. Metals in bone tissue of Antillean Manatees from the Gulf of Mexico and Chetumal Bay, Mexico. *Bull. Environ. Contam. Toxicol.* **2016**, *96*, 9–14, doi:10.1007/s00128-015-1674-6.
20. Rojas-Mingüer, A.; Morales-Vela, B. Metales pesados en hueso y sangre de manatíes (*Trichechus manatus manatus*) de la Bahía de Chetumal, Quintana Roo, México. In *Contribuciones de la Ciencia al Manejo Costero Integrado de la Bahía de Chetumal y su área de influencia*, 2nd ed.; Rosado-May, F.J., Romero-May, R., Navarrete, A., Eds.; Universidad de Quintana Roo: Chetumal, México, 2002; pp. 133–142; ISBN 968-7864-34-6.
21. Gajdosechova, Z.; Brownlow, A.; Cottin, N.T.; Fernandes, M.; Read, F.L.; Urgast D.S.; Raab, A.; Feldmann, J.; Krupp, E.M. Possible link between Hg and Cd accumulation in the brain of long-finned pilot whales (*Globicephala melas*). *Sci. Total Environ.* **2016**, *545*, 407–413, doi:10.1016/j.scitotenv.2015.12.082.
22. O'Hara, T.M.; Woshner, V.; Bratton, G. Inorganic pollutants in Artic marine mammals. In *Toxicology of Marine Mammals*, Vos, J.G., Bossart, G.D., Fournier, M., O`Shea, T.J., Eds.; Taylos & Francis New York, USA, 2003; Volume 3, pp. 206–246; ISBN 0-203-16557-8.
23. Zhang, X.; Lin, W.; Yu, R.-Q.; Sun, X.; Ding, Y.; Chen, H.; Chen, X.; Wu, Y. Tissue partition and risk assessments of trace elements in Indo-Pacific Finless Porpoises (*Neophocaena phocaenoides*) from the Pearl River Estuary coast, China. *Chemosphere* **2017**, *185*, 1197–1207, doi:10.1016/j.chemosphere.2017.07.080.
24. Sun, X.; Yu, R.-Q.; Zhang, M.; Zhang, X.; Chen, X.; Xiao Y.; Ding, Y.; Wu, Y. Correlation of trace element concentrations between epidermis and internal organ tissues in Indo-Pacific humpback dolphins (*Sousa chinensis*). *Sci. Total Environ.* **2017**, *605*, 238–245, doi:10.1016/j.scitotenv.2017.06.180.
25. Monteiro, S.S.; Pereira, A.T.; Costa, E.; Torres, J.; Oliveira, I.; Bastos-Santos, J.; Araújo, H.; Ferreira, M.; Vingada, J.; Eira, C. Bioaccumulation of trace element concentrations in common dolphins (*Delphinus delphis*) from Portugal. *Mar. Pollut. Bull.* **2016**, *113*, 400–407, doi:10.1016/j.marpolbul.2016.10.033.
26. Monteiro, S.S.; Torres, J.; Ferreira, M.; Marçalo, A.; Nicolau, L.; Vingada J.; Eira C. Ecological variables influencing trace element concentrations in bottlenose dolphins (*Tursiops truncatus*, Montagu 1821) stranded in continental Portugal. *Sci. Total Environ.* **2016**, *544*, 837–844, doi:10.1016/j.scitotenv.2015.12.037.

27. Reed, L.A.; McFeeW, E.; Pennington, P.L.; Wirth, E.F.; Fulton, M.H. A survey of trace element distribution in tissues of the dwarf spermwhale (*Kogia sima*) stranded along the South Carolina coast from 1990–2001. *Mar. Pollut. Bull.* **2015**, *100*, 501–506, doi:10.1016/j.marpolbul.2015.09.005.
28. Mahfouz, C.; Henry, F.; Courcot, L.; Pezeril, S.; Bouveroux, T.; Dabin, W.; Jauniaux, T.; Khalaf, G.; Amara, R. Harbour porpoises (*Phocoena phocoena*) stranded along the southern North Sea: An assessment through metallic contamination. *Environ. Res.* **2014**, *133*, 266–273, doi:10.1016/j.envres.2014.06.006.
29. Lemos, L.S.; De Moura, J.F.; Hauser-Davis, R.A.; De Campos, R.C.; Siciliano, S. Small cetaceans found stranded or accidentally captured in southeastern Brazil: Bioindicators of essential and non-essential trace elements in the environment. *Ecotoxicol. Environ. Saf.* **2013**, *97*, 166–175, doi:10.1016/j.ecoenv.2013.07.025.
30. Seixas, T.G.; Kehrig, H.A.; Beneditto, A.P.M.; Souza, C.M.M.; Malm, O.; Moreira, I. Essential (Se, Cu) and non-essential (Ag, Hg, Cd) elements: What are their relationships in liver of *Sotalia guianensis* (Cetacea, Delphinidae)? *Mar. Pollut. Bull.* **2009**, *58*, 601–634, doi:10.1016/j.marpolbul.2008.12.005.
31. Lavery, T.J.; Butterfield, N.; Kemper, C.M.; Reid, J.R.; Sanderson, K. Metals and selenium in the liver and bone of three dolphin species from South Australia, 1988–2004. *Sci. Total Environ.* **2008**, *390*, 77–85, doi:10.1016/j.scitotenv.2007.09.016.
32. Stavros, H.-C.W.; Bossart, G.D.; Hulsey, T.C.; Fair, P.A.; Trace element concentrations in blood of free-ranging bottlenose dolphins (*Tursiops truncatus*): Influence of age, sex and location. *Mar. Pollut. Bull.* **2008**, *56*, 348–379, doi:10.1016/j.marpolbul.2007.10.030.
33. Dehn, L.A.; Follmann, E.H.; Thomas, D.L.; Sheffield, G.G.; Rosa, C.; Duffy, L.K.; O'Hara, T.M. Trophic relationships in an Arctic food web and implications for trace metal transfer. *Sci. Total Environ.* **2006**, *362*, 103–123, doi:10.1016/j.scitotenv.2005.11.012.
34. Haynes, D.; Carter, S.; Gaus, C.; Muller, J.; Dennison, W. Organochlorine and heavy metal concentrations in blubber and liver tissue collected from Queensland (Australia) dugong (*Dugong dugon*). *Mar. Pollut. Bull.* **2005**, *51*, 361–369, doi:10.1016/j.marpolbul.2004.10.020.
